# Multiformin-Type Azaphilones Prevent SARS-CoV-2 Binding to ACE2 Receptor

**DOI:** 10.3390/cells12010083

**Published:** 2022-12-25

**Authors:** Linda Jansen-Olliges, Shambhabi Chatterjee, Lili Jia, Frank Stahl, Christian Bär, Marc Stadler, Frank Surup, Carsten Zeilinger

**Affiliations:** 1 Centre of Biomolecular Drug Research (BMWZ), Gottfried-Wilhelm-Leibniz Universität Hannover, Schneiderberg 38, 30167 Hannover, Germany; 2 Hannover Medical School, Institute of Molecular and Translational Therapeutic Strategies, OE 8886, Carl-Neuberg-Str. 1, 30625 Hannover, Germany; 3 Hubei International Scientific and Technological Cooperation Base of Traditional Fermented Foods, College of Food Science and Technology, Huazhong Agricultural University, Wuhan 430070, China; 4 Institute of Technical Chemistry, Gottfried-Wilhelm-Leibniz Universität Hannover, Callinstr. 5, 30167 Hannover, Germany; 5 Department Microbial Drugs, Helmholtz Centre for Infection Research GmbH (HZI), Inhoffenstraße 7, 38124 Braunschweig, Germany; 6 Institute of Microbiology, Technische Universität Braunschweig, Spielmannstraße 7, 38106 Braunschweig, Germany

**Keywords:** protein microarray, ACE2, SARS-CoV-2, protein-protein interaction

## Abstract

Protein microarray screenings identified fungal natural products from the azaphilone family as potent inhibitors of SARS-CoV-2 spike protein binding to host ACE2 receptors. Cohaerin F, as the most potent substance from the cohaerin group, led to more than 50% less binding of ACE2 and SARS-CoV-2 spike protein. A survey for structurally related azaphilones yielded the structure elucidation of six new multiformins E–J (**10**–**15**) and the revision of the stereochemistry of the multiformins. Cohaerin and multiformin azaphilones **(1–5, 8**, **12**) were assessed for their activity in a cell-based infection assay. Calu-3 cells expressing human ACE2 receptor showed more than 75% and 50% less infection by SARS-CoV-2 pseudotyped lentivirus particles after treatment with cohaerin C (1) and cohaerin F (**4**), respectively. Multiformin C (**8**) and G (**12**) that nearly abolished the infection of cells. Our data show that multiformin-type azaphilones prevent the binding of SARS-CoV-2 to the cell entry receptor ACE2.

## 1. Introduction

Coronaviruses are ubiquitously occurring and aroused worldwide attention as SARS, MERS, and SARS-CoV-2 (COVID-19), challenged the public health systems and led to tremendously negative consequences on the global economy [1,2,3]. Coronaviruses comprise a group of enveloped, positive-stranded RNA viruses infecting mammals and birds, while the original and intermediate hosts are still not known [4,5]. Besides the typical pedal-like spikes on their surface, they have a round shape with a very distinct morphology compared to other nidoviruses [6]. Earlier, it was described that human angiotensin–converting enzyme 2 (ACE2) is a target for SARS and NL63 coronaviruses [7,8].

In humans, coronaviruses cause respiratory infections, including the common cold. In other species, symptoms can vary: in chickens, they lead to upper respiratory diseases, while in cows and pigs, the symptoms include diarrhea. The viral entry in human cells is mediated by ACE2 as their receptor.

Additionally, ACE2 in humans has been associated with cardiovascular disorders, hypertension, and neurodegenerative diseases, which makes it a drug target with a relevant physiological key position [9,10,11,12,13].

Therefore, drug targeting of ACE2 can inhibit the activation of angiotensin I decapeptide into the vasoconstrictor angiotensin II octapeptide with compounds such as phosphinic peptides [14]. Recently, several structures of SARS-CoV-2 spike protein complexed with human ACE2 have been resolved (PDBids: 6LZG, 6VW1, 6M17), highlighting the critical residues involved in the intermolecular interactions between the viral spike protein and its receptor [15,16,17].

As the virus has been transmitted in humans from bats and/or other intermediate hosts, considerable work has been conducted to explore the sequence and structural variations in the spike proteins and animal host ACE2 receptors [17]. Although a high vaccination rate in the human population is the best protection, small molecule drugs against the virus are also required for several reasons. Different drug target sites of the virus can hinder its replication or cell entry, including the proteinase NSP5, the viral RNA, the helicase NSP13, the nucleocapsid (N), and the RBD of the spike protein. Several other drug sites which hinder the virus replication were found in the host. To shorten the clinical phase and costs, repurposing FDA/EMA-approved small molecules is also a strategy for finding inhibitors targeting, for example, the SARS-CoV-2 main protease [18,19].

In applying this strategy, several compounds with different targets were found. Viral protease inhibitors (nelfinavir, boceprevir), guanine analog (thioguanine), ionophoric antibiotics (salinomycin, monensin, maduramicin), functional inhibitors (cepharanthine, emetine, ivermectin, moxidectin, mefloquine) or ion channel modulators (ivacaftor, azelnidipine, penfluridol, dronedarone), often cause undesirable side effects [18,20,21,22,23,24,25].

Using a cell-based high-throughput screen, entry inhibitors of SARS-CoV-2, such as Imatinib, mycophenolic acid, and quinacrine dihydrochloride, were recently successfully tested in human cells and lung cell organoids [26]. Several studies have explained the variations in the coronavirus spike protein and host ACE2 receptor to predict and/or prove the origin and potential of cross-transmission of the virus [27]. Sixteen ACE2 variants are known, which can trace together with spike variations to higher susceptibility and infectivity [27,28]. For this reason, an efficient test system was developed based on a highly miniaturized protein microarray technique using fluorescent labeling to observe the binding of ACE2 receptors and spike protein. Subsequently, the microarray application was used to test compounds that inhibit the specific interaction of spike protein with ACE2 receptors, as there is a need for effective compounds to prevent the virus from entering cells. For this purpose, both already approved active substances and fungal natural products were tested. Particular interest was drawn to fungal products here, as numerous derivatives can be created by semi-synthesis or feeding approaches.

## 2. Materials and Methods

### 2.1. Materials

Cy5 Protein Labeling Kit was purchased from Jena Bioscience, Germany. A fragment of SARS-CoV-2 spike protein (Wuhan-Hu-1) with ACE2 binding activity was purchased from R&D systems (10500-CV) corresponding to accession no. YP_009724390.1 containing the sequence:

RVQPTESIVRFPNITNLCPFGEVFNATRFASVYAWNRKRISNCVADYSVLYNSASFSTFKCYGVSPTKLNDLCFTNVYADSFVIRGDEVRQIAPGQTGKIADYNYKLPDDFTGCVIAWNSNNLDSKVGGNYNYLYRLFRKSNLKPFERDISTEIYQAGSTPCNGVEGFNCYFPLQSYGFQPTNGVGYQPYRVVVLSFELLHAPATVCGPKKSTNLVKNKCVNF

Mycophenolic acid (MPA) (**16**), 4-[(4-Methylpiperazin-1-yl) methyl]-N- benzamide (Imatinib) (**17**), and 4-N-(6-chloro-2-methoxyacridin-9-yl)-1-N,1-N-diethylpentane-1,4-diamine; dihydrate; dihydrochloride (Quinacrine dihydrochloride dihydrate; QNHC) (**18**) purchased by Merck, Germany.

### 2.2. Preparation of Human ACE2

Human ACE2 cDNA was codon usage optimized, synthesized, and provided in a pET28a bacterial expression vector by (Synbio Technologies LLC, Monmouth Junction, NJ, USA) named hACE2.

### 2.3. Spectral Equipment

NMR spectra were recorded with an Avance III 700 spectrometer (Bruker BioSpin, Rheinstetten, Germany) with a 5 mm TCI cryoprobe (^1^H 700 MHz, ^13^C 175 MHz). NMR data were referenced to selected chemical shifts of acetone-*d*_6_ (^1^H: 2.05 ppm, ^13^C: 29.32 ppm), pyridine-*d*_5_ (^1^H: 7.22 ppm, ^13^C: 123.87 ppm) and CDCl_3_ (^1^H: 7.27 ppm, ^13^C: 77.00 ppm), respectively. Optical rotations were taken with an MCP 150 polarimeter (Anton Paar, Graz, Austria). UV/Vis spectra were taken with a UV-2450 UV/Vis spectrophotometer (Shimadzu, Kyoto, Japan), while ECD spectra were collected with a JD-815 spectrophotometer (Jasco, Pfungstadt, Germany). ESI-MS spectra were recorded with an UltiMate^®^ 3000 Series uHPLC (Thermo Fisher Scientific, Waltman, MA, USA) utilizing a C18 Acquity^®^ UPLC BEH column (2.1 × 50 mm, 1.7 µm; Waters, Milford, MA, USA) connected to an amaZon^®^ speed ESI-Iontrap-MS (Bruker). HPLC parameters were set as follows: solvent A: H_2_O + 0.1% formic acid, solvent B: acetonitrile (ACN) + 0.1% formic acid; gradient: 5% B (0.5 min), 5−100% (19.5 min), 100% (5 min), flowrate 0.6 mL/min, and DAD detection 190−600 nm. HR-ESI-MS spectra were obtained with an Agilent 1200 Infinity Series HPLC (Agilent Technologies, Böblingen, Germany; conditions same as for ESI-MS spectra) connected to a maXis^®^ ESI-TOF-MS (Bruker).

### 2.4. Isolation of Azaphilones

The azaphilones evaluated in the current study were obtained from stromata (fruiting bodies) of the ascomycetes *Jackrogersella cohaerens* and *J. multiformis.* These species had been previously classified in the genera *Hypoxylon* and *Annulohypoxylon* until the revision of the families of Xylariales by Wendt et al. [29] and now belong to the family Hypoxylaceae. The fungi were collected and identified by M. Stadler in the vicinity of Deidesheim, Germany, in September of 2021. Cohaerins C-G (**1–5**) were isolated in a similar manner as described previously [30] from the stromata of *Jackrogersella cohaerens* collected from *Fagus sylvatica* wood. For the isolation of multiformins **6–15**, dry stromata of *Jackrogersella multiforme* (16.62 g, collected from the wood of *Betula pendula*) were carefully detached from the woody substrate, transferred into the bottle, and extracted in an ultrasonic bath with 200 mL methanol at 40 °C for 30 min, filtered, and evaporated (40 °C ). After repeating this process twice, acetone was used for further extraction alike and repeated four times until the final extract became colorless. The combined organic extracts were suspended in water and extracted with ethyl acetate to get a crude extract (1.28 g). This crude extract was fractionated by normal-phase Flash chromatography (GRACE Reveleris X2 flash system) on a Reveleris 40 g silica cartridge (40 µm), using the gradient system (40 mL/min) A: n-heptane, B: dichloromethane, and C: acetone: 0–50% B in 5 min, 50–70% B in 15 min, 70–100% B in 5 min, 100% B in 5 min (AB system) and 0–30% C in 20 min, 30–60% C in 30 min, 60–100% C in 10 min, and 100% C in 10 min (BC system). The process was followed by UV absorption at 224 nm and yielded seven main fractions in which fractions #1–#3 and fraction #7 contained the targeted metabolites and were used for further separation. This separation was conducted by a Gilson reverse-phase HPLC system (Middleton, WI, USA) with a Nucleodur C18 column (250 × 21 mm, 5 µm). The column was eluted with an acetonitrile/water system (flow rate 20 mL/min). Fraction #1 was fractionated with a gradient from 35% to 55% acetonitrile in 50 min and to 100% in 10 min to get multiformin A at retention time (RT) 22.5 min, multiformin F at RT 30 min, multiformin B at RT 33 min, and multiformin C at RT 40 min. From fraction #2, multiformin E was obtained at RT 17.5 min with a gradient from 30% to 50% acetonitrile in 50 min. Multiformin H and G were isolated from fraction #3 on a gradient of 30–45% acetonitrile in 50 min at 15 min and 27 min, respectively. Multiformin I was obtained at RT 23 min from fraction #7 with a water/acetonitrile gradient increasing from 25% to 40% in 40 min and then to 100% in 15 min.

### 2.5. Spectral Data

Multiformin E (**10**): [α]^25^_D_ −66 (MeOH); CD (MeOH) λ_ext_ nm (Δ_ε_): 362 nm (+3.0); 327 nm (−6.5), 256 nm (+0.9), 224 nm (−2.0); UV (EtOH) λ_max_ (log ε): 353 nm (4.30); ^1^H NMR (700 MHz, acetone-*d*_6_: see Table 1; 13C NMR (175 MHz, acetone-*d*_6_): see Table 2; HR-ESI-MS *m*/*z* 429.2106 [M + H]^+^ (calculated for C_24_H_29_O_7_, 429.2106); Rt = 8.19 min.

Multiformin F (**11**): [α]^25^_D_ −53.0 (MeOH); CD (MeOH) λ_ext_ nm (Δ_ε_): 373 nm (+2.0), 328 nm (−5.7), 256 nm (+0.7), 201 nm (+2.9); UV (EtOH) λ_max_ (log ε): 357 nm (4.34), 200 nm (4.41); ^1^H NMR (700 MHz, acetone-*d*_6_: see Table 1; ^13^C NMR (175 MHz, acetone-*d*_6_): see Table 2; HR-ESI-MS *m*/*z* 409.1834 [M + H]^+^ (calculated for C24H25O6, 409.1834); Rt = 10.68 min.

Multiformin G (**12**): [α]^25^_D_ +290 (MeOH); CD (MeOH) λ_ext_ nm (Δ_ε_): 417 nm (+5.6), 352 nm (−12.0), 276 nm (+9.1), 256 nm (+7.5), 210 nm (−10.0); UV (EtOH) λ_max_ (log ε): 347 nm (4.20), 267 nm (4.32), 228 nm (4.32); ^1^H NMR (700 MHz, acetone-*d*_6_: see Table 1; ^13^C NMR (175 MHz, acetone-*d*_6_): see Table 2; HR-ESI-MS *m*/*z* 425.1786 [M + H]^+^ (calculated for C24H25O7, 425.1786); Rt = 8.59 min.

Multiformin H (**13**): [α]^25^_D_ +146 (MeOH); CD (MeOH) λ_ext_ nm (Δ_ε_): 367 nm (+3.1), 328 nm (−6.3), 210 nm (+2.9); UV (EtOH) λ_max_ (log ε): 356 nm (4.32), 229 nm (4.23); ^1^H NMR (700 MHz, acetone-*d*_6_: see Table 1; ^13^C NMR (175 MHz, acetone-*d*_6_): see Table 2; HR-ESI-MS *m*/*z* 427.1942 [M + H]^+^ (calculated for C24H27O7, 427.1942); Rt = 7.82 min.

Multiformin I (**14**): [α]^25^_D_ +32 (MeOH); CD (MeOH) λ_ext_ nm (Δ_ε_): 360 nm (+5.1), 320 nm (−5.9), 257 nm (+1.0), 226 nm (−1.1), 214 nm (−0.07); UV (EtOH) λ_max_ (log ε): 350 nm (4.31), 206 nm (4.24); ^1^H NMR (700 MHz, acetone-*d*_6_: see Table 1; ^13^C NMR (175 MHz, acetone-*d*_6_): see Table 2; HR-ESI-MS *m*/*z* 403.2116 [M + H]^+^ (calculated for C23H31O6, 403.2116); Rt = 7.34 min.

Multiformin J (**15**)**:** UV (AcCN/H_2_O) λ_max_: 360 nm, 220 nm; ^1^H NMR (700 MHz, CHCl_3_-*d*: see Table 1); ^13^C NMR (175 MHz, CHCl_3_-*d*): see Table 2; ESI-MS *m*/*z* 405.11 [M + Na]^+^, 383.12 [M + H]^+^, 365.11 [M + H-H_2_O]^+^; HR-ESI-MS *m*/*z* 383.1854 [M + H]^+^ (calculated for C_23_H_27_O_5_, 383.1853); Rt = 10.7 min.

### 2.6. Mosher’s Analysis of Multiformin E (***10***)

For the preparation of the (*S*)-MTPA ester, 0.5 mg of multiformin E **10** was dissolved in 250 µL of pyridine-*d*_5_, and 10 µL of (*R*)-MTPA chloride was added. The mixture was incubated at ambient temperature for 30 min, and ^1^H NMR and ^1^H/^1^H TOCSY were measured. ^1^H NMR (700 MHz, pyridine-*d*_5_): similar to **9**, but *δ*_H_ 5.65 (13–H), 3.22 (12–H_a_), 2.95 (12–H_b_), 2.34 (14-H_a_), 2.24 (15-H), 1.73 (14-H_b_), 0.95 (16-H_3_) ppm. The (*R*)-MTPA ester was prepared in the same manner by the addition of 10 µL of (*S*)-MTPA chloride: ^1^H NMR (700 MHz, pyridine-*d*_5_): similar to **10**, but *δ*_H_ 5.65 (13–H), 3.03 (12–H_a_), 2.79 (12–H_b_), 2.35 (15-H), 2.28 (14-H_a_), 1.80 (14-H_b_), 1.19 (16-H_3_) ppm.

### 2.7. Expression and Purification of Human ACE2

The plasmid was transfected into *E. coli* BL21(DE3) cells and precultures were prepared. The next day main cultures (Terrific Broth) were inoculated with precultures (1:50) and incubated at 37 °C for 6 h. The cell suspension was cooled to 8 °C, induced with 1 mM isopropylthiogalactoside (IPTG, Carl Roth, Karlsruhe, Germany), and maintained at 8 °C for 70 h. After harvesting by centrifugation (8500× *g*, 15 min), the cells were either stored at −80 °C or directly resuspended in lysis buffer (20 mM Tris-HCl, pH 8.0, 50 mM KCl, 2 mM β-mercaptoethanol, 2 mM imidazole, 0,05% Tween-20, 10% glycerol) and lysed twice with a French press using 14,000–16,000 p.s.i.. Cell lysates were incubated with 2.5% glycerol, 2.5% PEG400, 1% N-lauroyl sarcosine, and 5 M urea for one hour at 37 °C and subsequently centrifuged at 20,000× *g* and 4 °C for one hour. A Ni-Indigo column (Cube Biotech, Monheim, Germany) was equilibrated with buffer A (20 mM Tris-HCl pH 8.0, 50 mM KCl, 0,05% Tween-20, 2 mM β-mercaptoethanol, 10% glycerol) and supernatant (1:2 diluted with lysis buffer) was loaded on the column. Hexa-histidine-tagged (6x-His) human ACE2 was eluted with 500 mM imidazole in buffer A and further purified by size-exclusion chromatography using buffer A and concentrated to 3 mg/mL using Amicon centrifugal spin filters (30K) in storage buffer (20 mM Tris-HCl, pH 7.5, 50 mM KCl, 2 mM β-mercaptoethanol, 10% (*v*/*v*) glycerol). The presence and purity of the protein were detected by SDS-PAGE and immune blotting using mAb anti-His antibody (antikoerper-online.de, Aachen, Germany) and secondary anti-mouse alkaline phosphatase (Merck, Darmstadt, Germany).

### 2.8. Cy5 labeling of ACE2 Protein

Purified full-length ACE2 was labeled with Cy5 using the Cy5 protein labeling kit (Jena Bioscience, Jena, Germany) following the manufacturer’s instructions. Free dye was removed by dialysis against 20 mM Tris-HCl pH 7.5, 50 mM KCl, and 10% glycerol.

### 2.9. Protein Microarray Detecting ACE2 and Spike Protein Interaction

Spike protein (2 mg/mL in 20 mM Tris-HCl, pH 7.5, 50 mM KCl, 2 mM β-mercaptoethanol, 10% (*v*/*v*) glycerol) was spotted on UniSart^®^ 3D nitro slides (Sartorius Stedim Biotech S.A., Göttingen, Germany) using a contactless GeSim Nano-PlotterTM (GeSim, Radeberg, Germany) with a nanotip pipette. After spotting, the chip surface was blocked with 1% BSA in buffer B (20 mM Tris-HCl, pH 7.5, 50 mM KCl, 2 mM β-mercaptoethanol) for 45 min and washed three times with buffer B. The chip was incubated at 4 °C for 16 h with 100 µM labeled ACE2 and the compounds of interest at varying concentrations. After washing three times with buffer B, the microarray was scanned using a GenePix 4000B Laser Scanner (Molecular Devices, Inc., San Jose, CA, USA) and 635 nm excitation wavelength. Quantification was done using the GenePix Pro 6 software. The dose-response curves for the binding of ACE2 and spike protein were calculated with GraphPad Prism 9 (San Diego, CA, USA).

### 2.10. Analysis of the Antiviral Activity of Compounds on Spike-GFP Production

Human Calu-3 lung epithelial cells were seeded in a 96-well black plate in DMEM containing 10% FBS for 24 h (37 °C and 5% CO_2_). SARS-CoV-2 spike B.1.1.7 pseudo virus encoding the fluorescent protein ZsGreen was produced as per the manufacturer’s protocol (Takara, #631295). The next day, cells were pretreated for 16 h with compound or vehicle control (DMSO) in 50 μL of serum-free OptiMEM and subsequently infected with SARS-CoV-2 spike B.1.1.7 pseudoparticles (2.45 × 10^5^ PFU/mL). At 48 h post-infection, cells were washed once and stored in PBS after fixation, and automated microscopy was performed using a Cytation device (Agilent, Santa Clara, CA, USA) in the 4X objective (eight fields per well, covering the entire well). Images were analyzed using the Analysis Module of the Cytation (Agilent, Santa Clara, CA, USA)) device to identify the percentage of GFP-positive cells in each well. The percentage of GFP-positive cells for experimental conditions were normalized against the control (untreated Calu-3 cells infected with the SARS-CoV-2 spike pseudoparticle) cells and expressed as relative infectivity. Three biological replicates were performed.

## 3. Results

**Scheme 1:**
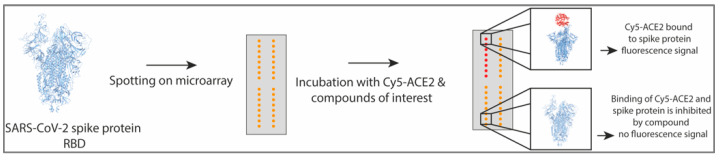
Workflow of protein microarray experiments. SARS-CoV-2 spike protein is spotted on nitrocellulose-coated glass chips after blocking incubated with Cy5-ACE2 and compounds of interest. An increase in fluorescence signal shows the binding of Cy5-ACE2 to the spotted spike protein. Binding inhibition is detected by lower/no fluorescence signal at positions of the spotted spike protein.

A highly miniaturized protein microarray assay was established to study spike protein-ACE2 interaction. Human ACE2 was recombinantly produced and purified by Ni-INDIGO-IMAC, followed by size exclusion chromatography (SEC). The purified protein was labeled with Cy5 for detection in interaction assays. Spike protein, containing the RBD domain, was spotted at a concentration of 2 mg/mL on a nitrocellulose-coated glass slide. After blocking, the blocks were incubated with different concentrations of Cy5 labeled ACE2. The binding activity of spotted spike protein and ACE2 was monitored by Cy5 fluorescence intensity at the positions of spike protein on the chip (Scheme 1).

**Figure 1:**
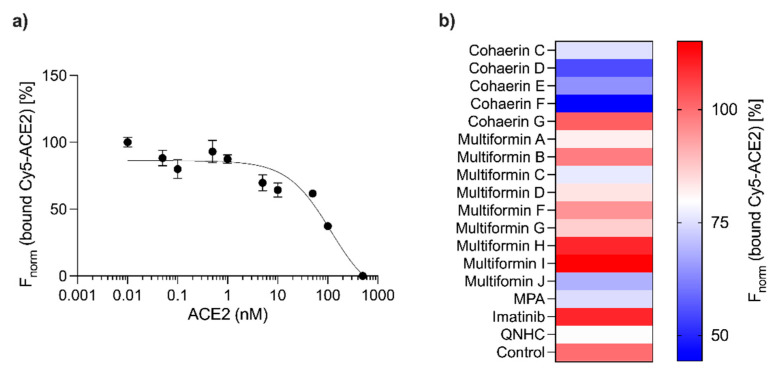
The development of a microarray-based binding assay for ACE2 and spike protein. (**a**) Displacement of Cy5-ACE2 by unlabeled ACE2 from spike protein. Normalized fluorescent intensity (F_norm_ (bound Cy5-ACE2) [%]) as displacement data fitted by a dose-response curve and EC_50_ of 115 nM. (**b**) Heat map showing binding inhibition of ACE2 and spike protein by substances **1–17** Binding of Cy5-ACE2 to spotted spike protein in the presence of 40 µM of the compounds. The fluorescence intensity is given in percentage compared to Cy5-ACE2 binding in control without substance.

The displacement of labeled ACE2 by unlabeled ACE2 proteins resulted in an affinity of 115 nM towards spike protein (Figure 1a). 100 nM Cy5 labeled ACE2 was used for further screenings. For establishing the screening assay, published SARS-CoV-2 inhibitors were tested [26]. In the initial screen, mycophenolic acid (MPA) (**16**) and quinacrine dihydrochloride (QNHC) (**18**) resulted in reduced binding activity at 40 µM concentration. In contrast, Imatinib (**17**) showed no displacement of ACE2 from spike protein (Figure 1b).

Next, we tested fungal natural products and drugs that are FDA approved for other purposes for their ability to influence the spike-ACE2 interaction (Figure 1b, Figures S21 and S22, Tables S1 and S2). The most potent activities were obtained by natural products of the azaphilone group, isolated and described recently as cohaerins from the Xylariaceous fungus *Jackrogersella cohaerens* [30,31]. All cohaerins tested (**1–5**) showed more than 25% binding inhibition at a concentration of 40 µM, with cohaerin F (**4**) showing the strongest displacement of ACE2 (56%) (Figure 1b).

**Figure 2:**
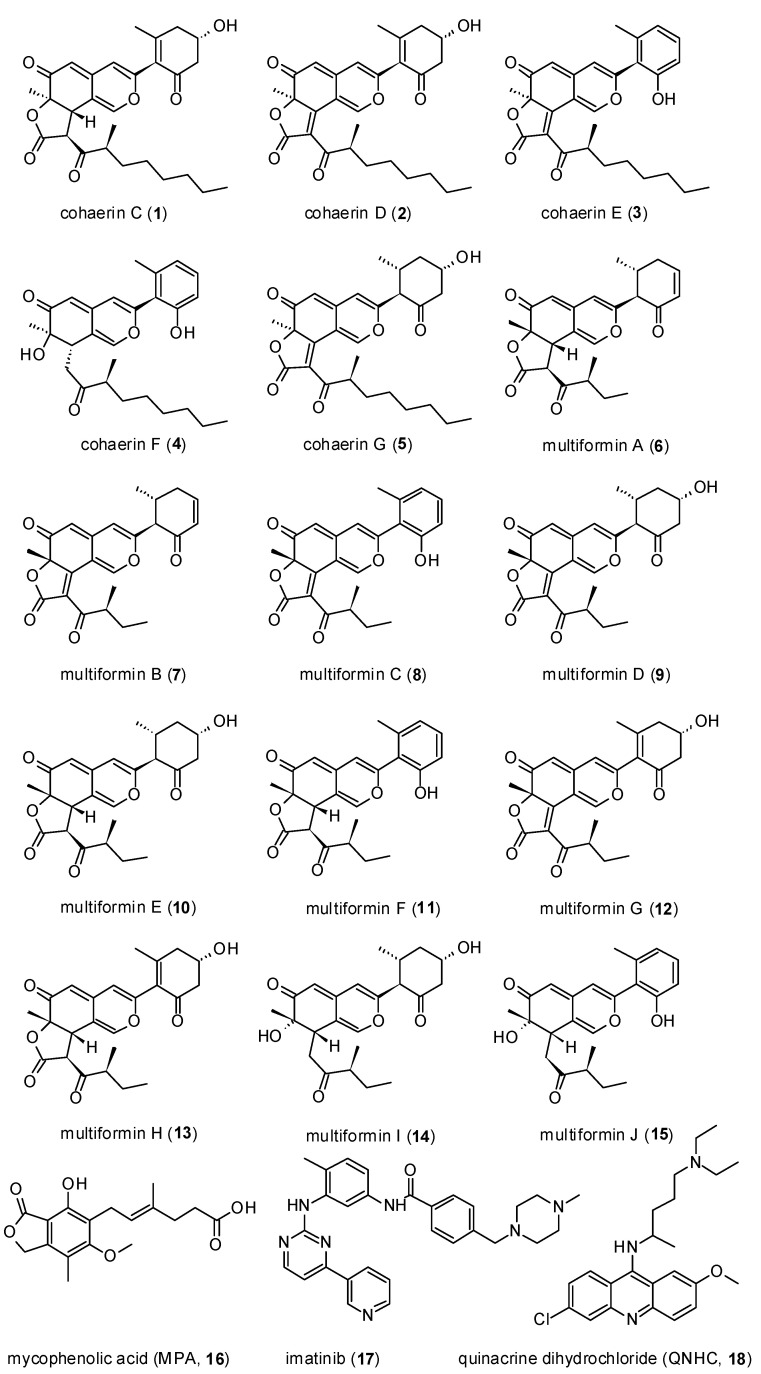
Selected compounds were tested in microarray-based displacement assay.

The identification of cohaerin F (**4**) as a potent inhibitor of the interaction of ACE2 with SARS-CoV-2 spike protein led us to investigate structurally closely related azaphilones, including cohaerins C-E, G (**1–3, 5**) and multiformins A (**6**)–D (**9**). In the course of the reisolation of **6–9**, new derivatives **10–15** were obtained by preparative HPLC. Their molecular formulas C_24_H_28_O_7_, C_24_H_24_O_6_, C_24_H_24_O_7_, C_24_H_26_O_7_, C_23_H_30_O_6,_ and C_23_H_26_O_5,_ were indicated by quasimolecular ion peak clusters at *m/z* 429.2106, 409.1834, 425.1786, 427.1942, 403.2116 and 383.12, respectively. The structures, elucidated by 1D and 2D NMR data (Figure 2, Table 1 and Table 2, Figures S1–S20), show mainly individual combinations of the variations of the six-membered ring moiety, sp^3^ or sp^2^ hybridization of C–8/C–18 and decarboxylation of C–17, which was observed analogously for the cohaerins and other azaphilones [29,30]. The new derivatives **10–15** were named multiformins E–J. Furthermore, a series of experiments were conducted with multiformin E (**10**) to determine the unknown stereochemistry of the multiformins. Briefly, the ΔδSR chemical shift pattern with positive ones for 12–H_a_ & 12–H_b_ and negative values for 14–H_a_, 14–H_b_, 15–H & 16–H_3_ of **10**–MTPA derivatives indicated a 13S configuration [32]. The 7S configuration was assigned upon CD data, a method confirmed by total synthesis [33]. ROESY correlations between 9–H_3_ and 8–H, but not between 9–H_3_ and 18–H were observed. Between 8–H and 18–H, a TOCSY-type artifact was observed, which was probably misassigned as a real correlation in the original description of multiformin A [33,34]. Consequently, the configuration of the multiformins was revised to 8R,18S. Finally, the resonances of C-21 (δ_C_ 25.2/δ_H_ 1.74, 1.31) and C-23 (δ_C_ 16.0/δ_H_ 1.06) indicated a 20S configuration [35].

**Figure 3:**
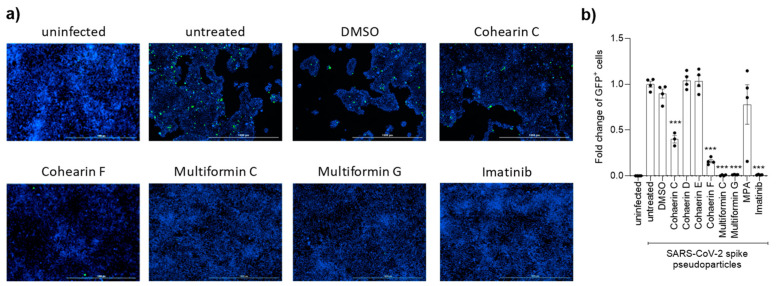
Antiviral activity of selected substances in cell-based infection assay. (**a**) Representative images of Calu-3 cells infected with SARS-CoV-2 spike pseudoparticle in the absence or presence of cohaerin C, cohaerin F, multiformin C, multiformin G and imatinib treatment. DMSO is used as a treatment control. Scale bar 1000 µm. (**b**) The quantification of GFP-positive cells (represented in fold change with respect to untreated Calu-3 cells infected with the SARS-CoV-2 spike pseudoparticle). Infection in Calu-3 cells is significantly reduced in the presence of cohaerin C, cohaerin F, multiformin C, multiformin G and imatinib (*n* = 3–4 wells per group). ∗∗∗ *p* < 0.001; one-way ANOVA, Tukey multiple-comparisons test.

To further characterize the natural products, the antiviral activity of the cohaerins **1–5**, multiformins C and G (**8**, **12**) and **16–17** as known inhibitors was analyzed on Calu-3 cells expressing human ACE2 receptor proteins. Here we produced SARS-CoV-2 spike pseudovirus encoding the fluorescent protein ZsGreen. Their binding with human ACE2 was analyzed in the presence and absence of substances. The representative images (Figure 3a) include only the selected compounds which showed the most significant effect after compound treatment. The treatment with 100 µM cohaerin F (**4**) resulted in 75% less infection, whereas 100 µM cohaerin C (**1**) inhibited 50% infection of the cells (Figure 3b). 

Compounds **2**, **3**, and **5** showed toxic effects on the Calu-3 cells. In the case of the latter, no evaluation of results due to the low number of living cells was possible. The number of GFP-positive cells was decreased to nearly zero after treatment with 2.4 µM **8** and **12** (Figure 3). **17** as the most potent compound inhibited the SARS-CoV-2 cell entry at a concentration of 1 µM.

## 4. Discussion

We transferred a recombinantly synthesized and purified ACE2 receptor to a highly miniaturized microarray-based approach to monitoring the interaction with the spike protein. Recently, antiviral activity against SARS-CoV-2 of compounds **16–18** was described, and their ability to inhibit spike protein-ACE2 interaction was tested in our assay. Han et al. investigated the mechanism of infection blocking, and their SPR binding analysis suggested that Imatinib and QNHC bind to ACE2, whereas MPA and QNHC treatment additionally decreased furin expression levels [26]. In our microarray assay, **16** and **18** are successful in displacing ACE2 from spike protein. In contrast, in the cell-based infection assay, **17** is the most active compound. The different approaches to mimic and simplify an *in vivo* happening process can give divergent results based on the methods or conditions used. *In vitro* assays like the ones used in our study can help give an idea about certain processes but cannot completely represent the complex native environment. The screening of the natural product library identified substances of the cohaerin and multiformin group from the fungi *Jackrogersella cohaerens* and *J. multiformis* that were particularly striking in significantly reducing the binding of ACE2 and spike protein. In addition, cell-based assays with Calu-3 cells have shown that in the presence of **1**, **4**, **8**, and **12,** viral entry is drastically reduced. It should be noted that for *in vitro* screens, only the spike RBD was used, whereas, in the cell-based assay, the full-length spike protein was used. Whether and how the binding to the proteins are directly influencing the interaction with the other binding partner or is causing structural changes is not clear. That question should be addressed in the following studies.

Compounds of the cohaerin and multiformin groups showed high potency and can serve as lead compounds for semi-synthetic approaches to creating derivatives. Small molecules can function as an additional tool to vaccinations by preventing and containing pandemic outbreaks like SARS-CoV-2 because of their easy application, cost-effective fabrication, and convenient handling.

## 5. Conclusions

We isolated seven new multiformin azaphilones and assigned the stereochemistry of all stereocenters. Using a highly miniaturized microarray-based displacement assay with Cy5-tagged ACE2 protein, compounds that abolish the binding of ACE2 to spotted spike protein were identified. Additionally, cohaerin and multiformin azaphilones were found to inhibit binding in a cell-based infection assay.

## Figures and Tables

**Table 1:** ^1^H NMR data (700 MHz) of multiformins E-K (**10–15**) in acetone-d_6_.

POS	10	11	12	13	14	15 ^a^
**1**	7.36, d (0.9)	7.46, s	8.75, d (1.3)	7.38, d (1.3)	6.61, s	7.43, br s
**4**	6.26, s	6.47, s	6.41, s	6.24, s	6.21, d (2.8)	6.27, m
**5**	5.3, d (1.3)	5.42, s	5.35, d (0.86)	5.35, d (1.3)	5.29, m	5.44, s
**8**	3.91, d (12.1)	3.98, d (12.1)		3.94, d (12.1)	3.32, m	3.47, m
**9**	1.51, s	1.56, s	1.68, s	1.53, s	1.21, s	1.36, s
**10**	3.32, d (12.1)				3.27, m	
**12**	2.63, ddd (13.4, 5.0, 1.9)	6.85, d (8.2)	2.73, dt (15.9, 1.8)	2.69, dd (15.9, 4.0)	2.62, m	6.75, d (7.9)
	2.48, dd (13.4,11.6)		2.541 ddd (15.9, 7.7, 0.9)	2.50, ddd (15.9, 8.0, 0.9)	2.46, m	
**13**	3.92, m	7.19, m	4.34, ddt (7.8, 6.1, 4.0)	4.30, ddt (8.0, 5.4, 4.0)	3.91, m	7.20, t (7.9)
**14**	1.60, m	6.79, s	2.63, dd (18.5, 6.5)	2.86, m	2.23 dtd (13.0, 3.8, 2.2)	6.82, d (7.9)
	2.23, m		2.91, m	2.59, m	1.59, m	
**15**	2.11, m				2.08, m	
**16**	1.04, d (6.5)	2.22, m	2.11, m	2.02, br s	1.02, d (6.5)	2.22, s
**18**	4.41, d (12.1)	4.47, d (12.1)		4.43, d (12.1)	2.85, m	3.02, m
					2.49, m	2.51, m
**20**	2.95, dqd (7.3, 7.2, 6.0)	2.96, m	3.50, sxt (6.9)	2.95, m	2.39, m	2.33 m
**21**	1.74, ddq (14.0, 7.3, 7.2)	1.75, m	1.82, m	1.74, m	1.59, m	1.72, m
	1.38, dqd (14.0, 7.3, 6.0)	1.38, m	1.35, dqd (13.5, 7.3, 6.9)	1.37, m	1.29, m	1.36, m
**22**	0.87, t (7.3)	0.87, t (7.5)	0.94, t (7.3)	0.87, t (7.5)	0.82, t (7.3)	0.87, d (7.5)
**23**	1.06, d (7.3)	1.06, d (7.3)	1.00, d (6.9)	1.05 d (7.3)	0.95, d (7.0)	0.96, d (6.9)

^a^ measured in CDCl_3._

**Table 2:** ^13^C NMR data (175 MHz) of multiformins E-J (**10–15**) in acetone-d_6_.

POS	10	11	12	13	14	15 ^a^
**1**	147.6, CH	148.1, CH	154.3, CH	147.9, CH	145.4, CH	147.0, CH
**3**	159.2, C	156.7, C	154.7, C	155.3, C	158.7, C	154.7, C
**4**	110.9, CH	112.2, CH	113.7, CH	112.3, CH	110.8, CH	111.6, CH
**5**	106.7, CH	106.9, CH	106.3, CH	107.1, CH	104.0, CH	104.4, CH
**6**	191.5, C	191.5, C	190.5, C	191.5, C	198.6, C	198.8, C
**7**	83.5, C	83.7, C	88.4, C	83.5, C	73.6, C	73.2, C
**8**	44.8, CH	44.5, CH	167.0, C	44.8, CH	42.0, CH	40.8, CH
**9**	23.2, CH_3_	23.3, CH_3_	26.2, CH_3_	23.3, CH_3_	27.0, CH_3_	26.9, CH_3_
**10**	61.7, CH	120.8, C	130.6, C	131.1, C	61.7, CH	119.6, C
**11**	204.2, C	156.5, C	194.6, C	194.7, C	204.2, C	153.8, C
**12**	51.4, CH_2_	114.3, CH	46.9, CH_2_	46.9, CH_2_	51.4, CH_2_	113.8, CH
**13**	68.2, CH	131.8, CH	65.6, CH	65.6, CH	68.2, CH	131.3, CH
**14**	43.4, CH_2_	122.1, CH	41.8, CH_2_	41.7, CH_2_	43.4, CH_2_	122.9, CH
**15**	31.6, CH	139.4, C	162.5, C	161.7, C	31.5, C	138.8, C
**16**	20.5, CH_3_	19.7, CH_3_	22.9, CH_3_	23.2, CH_3_	20.5, CH_3_	19.9, CH_3_
**17**	170.3, C	170.4, C	169.0, C	170.3, C	-	-
**18**	55.7, CH	55.8, CH	123.9, C	55.7, CH	39.9, CH_2_	39.0, CH_2_
**19**	207.2, C	207.2, C	201.1, C	207.2, C	213.1, C	213.5, C
**20**	48.5, CH	48.6, CH	45.5, CH	44.5, CH	49.1, CH	48.7, CH
**21**	25.2, CH_2_	25.2, CH_2_	25.9, CH_2_	25.2, CH_2_	26.1, CH_2_	25.7, CH_2_
**22**	11.9, CH_3_	11.9, CH_3_	12.0, CH_3_	11.9, CH_3_	11.9, CH_3_	11.7, CH_3_
**23**	16.0, CH_3_	15.9, CH_3_	16.2, CH_3_	15.9, CH_3_	15.9, CH_3_	15.7, CH_3_
**4A**	144.3, C	144.8, C	144.5, C	144.4, C	147.0, C	144.9, C
**8A**	115.5, C	115.5, C	111.9, C	115.5, C	121.2, C	120.6, C

^a^ measured in CDCl_3._

## Data Availability

Data availability statements are available upon publication.

## Supplementary Materials

The following are available online at https://www.mdpi.com/article/10.3390/cells12010083/s1, Figure S1: HPLC-UV/vis chromatograms of crude extracts of *Jackrogersella multiformis* at 210 nm; Figure S2: HPLC-UV/Vis chromatograms at 210 nm and HR-ESI-MS(+) spectra of multiformins E-I (**10–14**); Figure S3: ECD spectra of multiformins E-I; Figure S4: COSY (blue arrows), HMBC (green arrows) and ROESY (violet arrows) correlations, indicating the structures of multiformins E (**10**)—J (**15**); Figure S5: ^1^H NMR spectrum (700 MHz, acetone-*d*_6_) of multiformin E (**10**); Figure S6: ^13^C NMR spectrum (175 MHz, acetone-*d*_6_) of multiformin E (**10**); Figure S7: COSY NMR spectrum (700 MHz, acetone-*d*_6_) of multiformin E (**10**); Figure S8: HSQC NMR spectrum (700 MHz, acetone-*d*_6_) of multiformin E (**10**); Figure S9: HMBC NMR spectrum (700 MHz, acetone-*d*_6_) of multiformin E (**10**); Figure S10: ROESY NMR spectrum (700 MHz, acetone-*d*_6_) of multiformin E (**10**); Figure S11: ^1^H NMR spectrum (700 MHz, acetone-*d*_6_) of multiformin F (**11**); Figure S12: ^13^C NMR spectrum (175 MHz, acetone-*d*_6_) of multiformin F (**11**); Figure S13: ^1^H NMR spectrum (700 MHz, acetone-*d*_6_) of multiformin G (**12**); Figure S14: ^13^C NMR spectrum (175 MHz, acetone-*d*_6_) of multiformin G (**12**); Figure S15: ^1^H NMR spectrum (700 MHz, acetone-*d*_6_) of multiformin H (**13**); Figure S16: ^13^C NMR spectrum (175 MHz, acetone-*d*_6_) of multiformin H (**13**); Figure S17: ^1^H NMR spectrum (700 MHz, acetone-*d*_6_) of multiformin I (**14**); Figure S18: ^13^C NMR spectrum (175 MHz, acetone-*d*_6_) of multiformin I (**14**); Figure S19: ^1^H NMR spectrum (700 MHz, CDCl_3_-*d*) of multiformin J (**15**); Figure S20: ^13^C NMR spectrum (175 MHz, CDCl_3_-*d*) of multiformin J (**15**); Figure S21: Compounds tested in the microarray-based assay; Figure S22: Heat map showing binding inhibition of ACE2 and spike protein by substances **19**–**31**; Table S1: Cytotoxicity of cohaerins (**1**–**5**) and multiformins (**6**–**15**) tested on mouse fibroblast L-929 cancer cells and KB3.1 ACC 158 cells; Table S2: All tested substances in the microarray-based screening assay.
