# Supplementary material for: Multiformin-Type Azaphilones Prevent SARS-CoV-2 Binding to ACE2 Receptor
Source: Cells. 2022 Dec 25;12(1):83. doi: 10.3390/cells12010083 (PMC9818685; doi:10.3390/cells12010083)
Supplement: Supplementary file 1 [file cells-12-00083-s001.zip › cells-1828244-supplementary.pdf]

Supplementary Material:

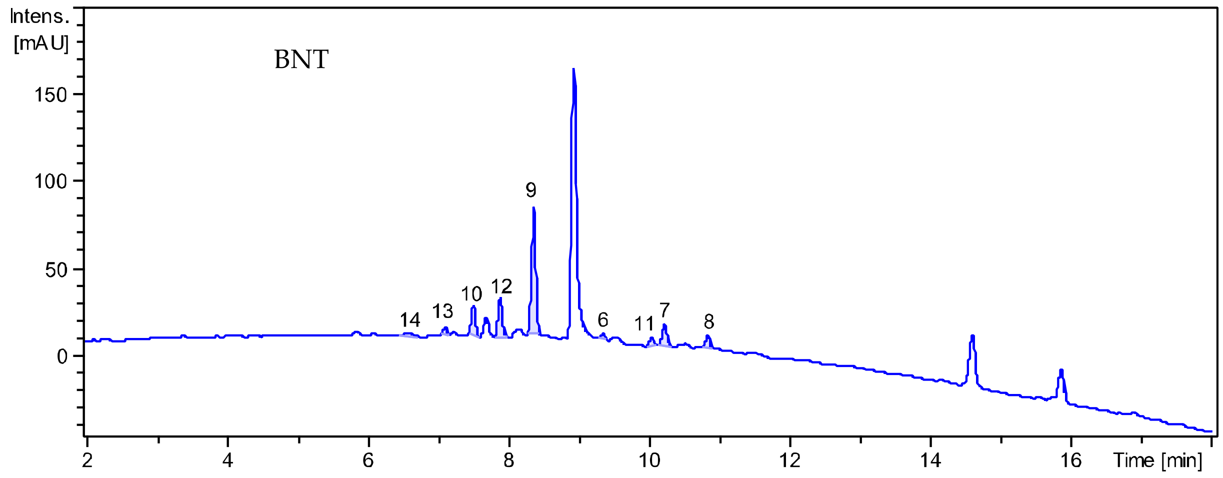

Figure S1: HPLC-UV/vis chromatograms of crude extracts of *Jackrogersella multiformis* at 210 nm. Peaks for multiformins A (**6**) - multiformin I (**13**) are marked with numbers.

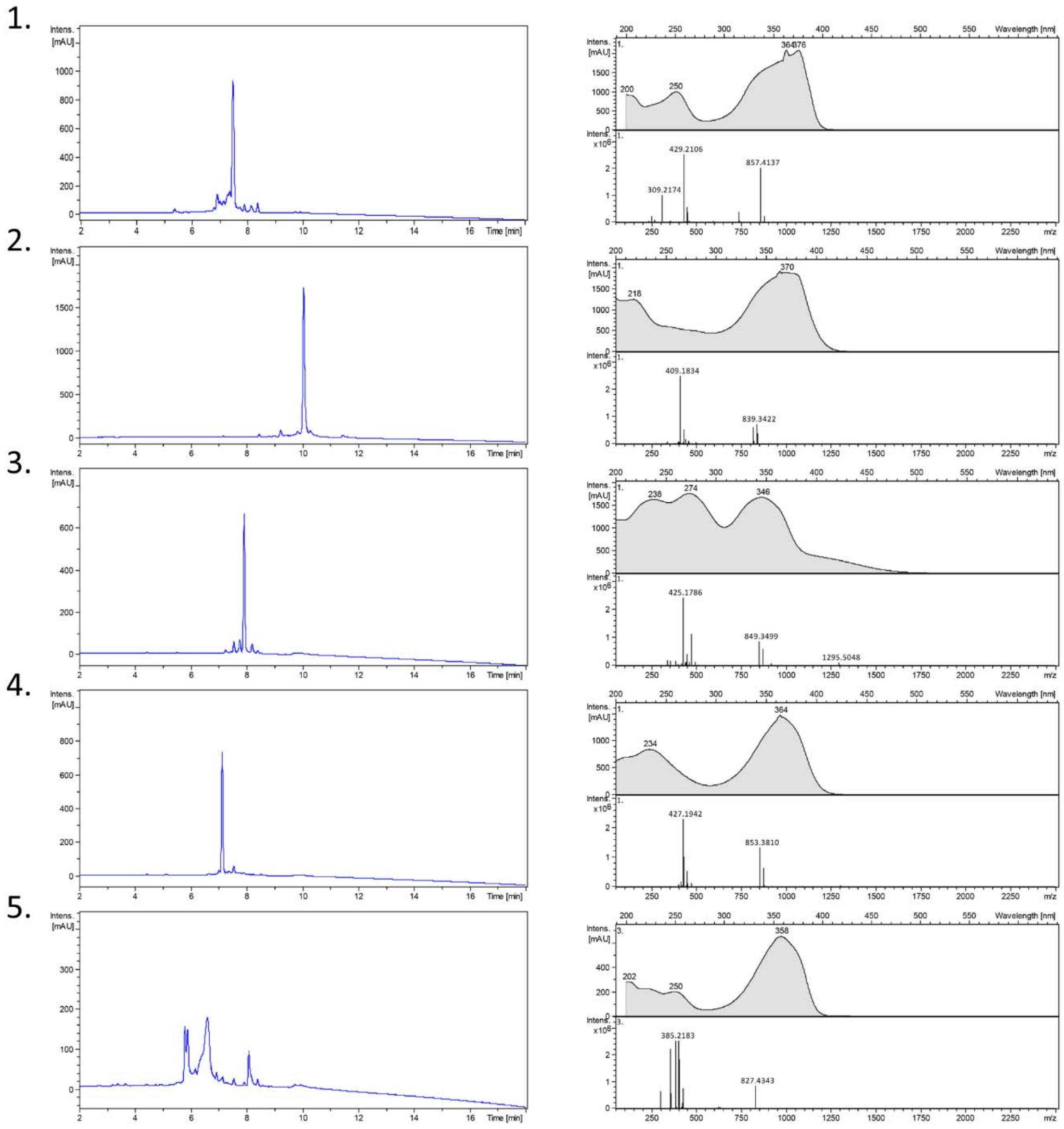

Figure S2. HPLC-UV/Vis chromatograms at 210 nm and HR-ESI-MS(+) spectra of multiformins E-I (**9**-**13**).

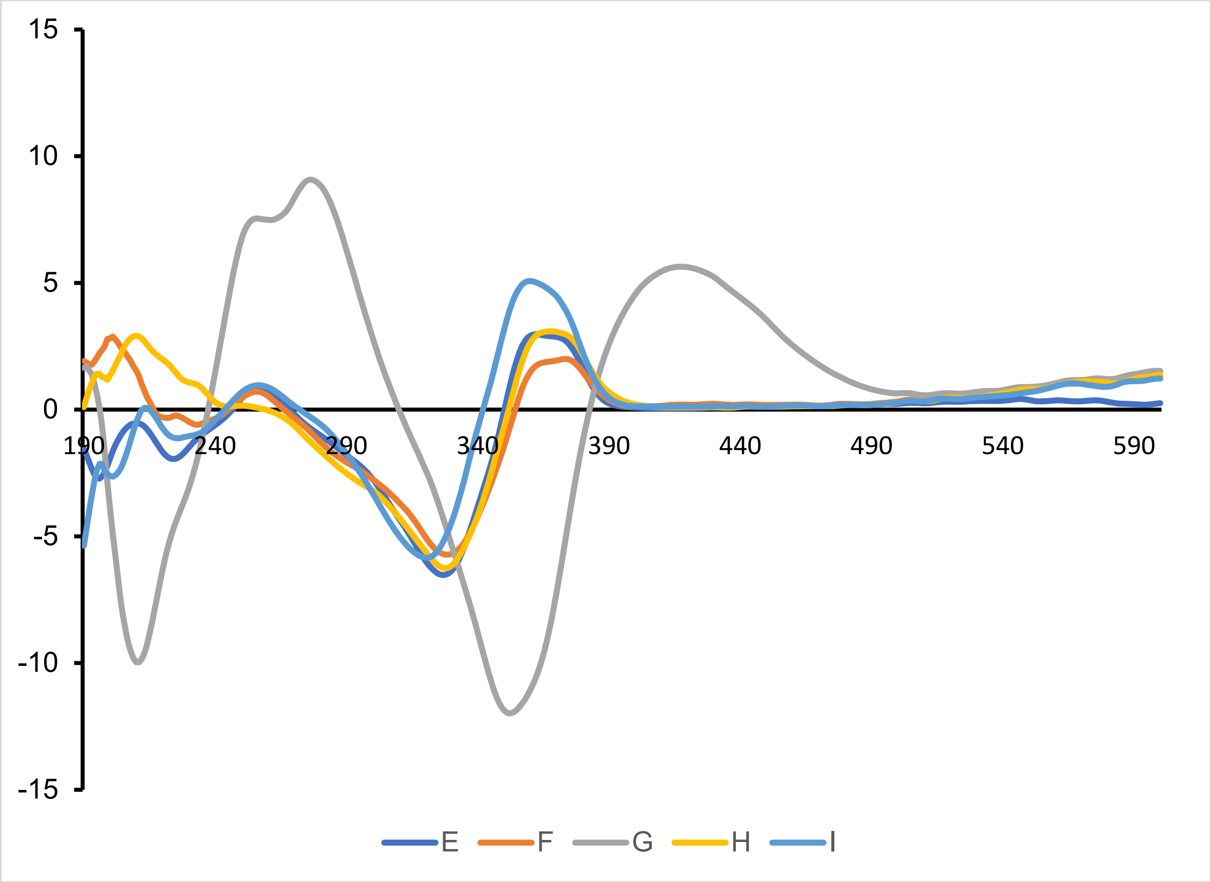

Figure S3. ECD spectra of multiformins E-I in methanol from 190 nm – 600 nm.

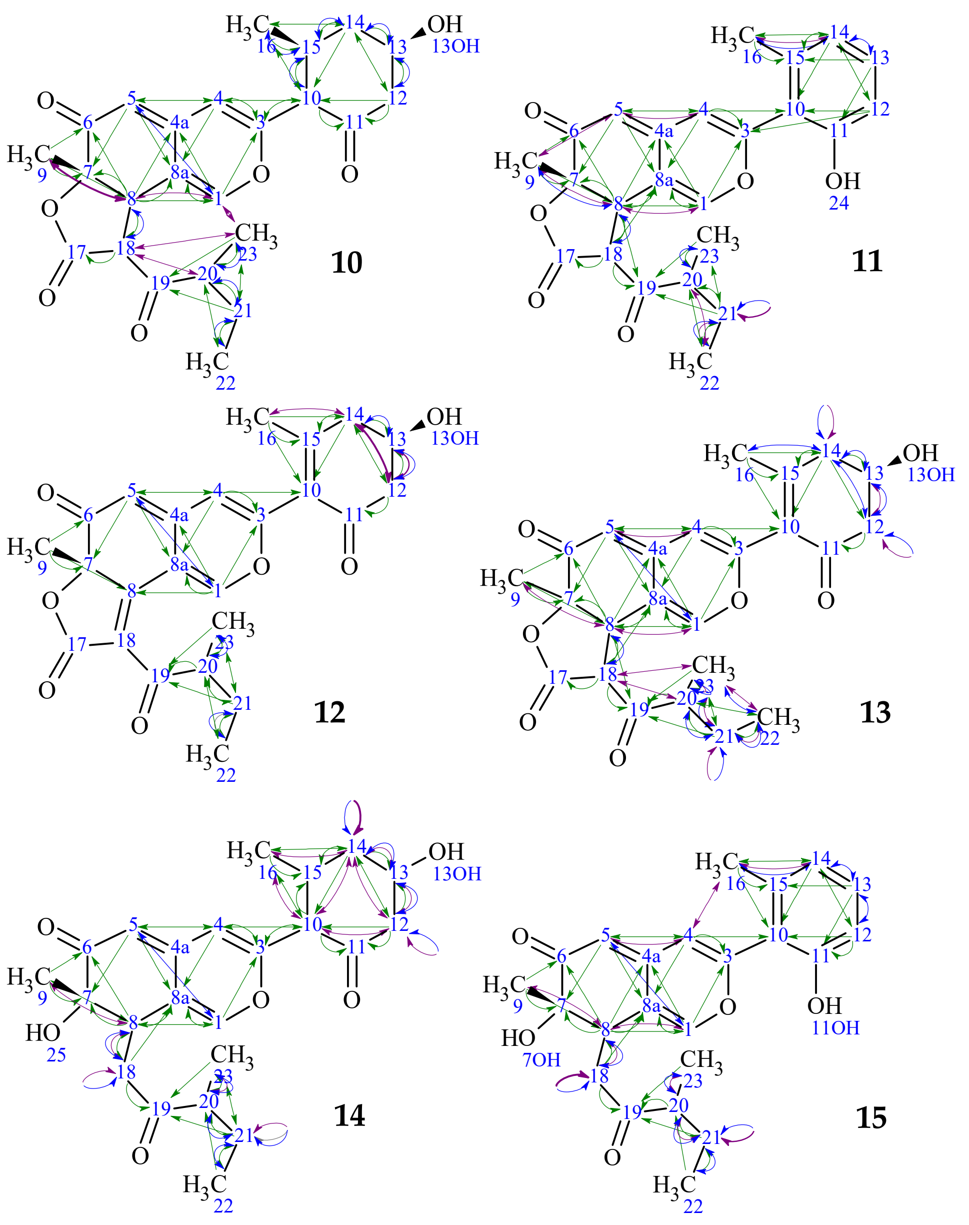

Figure S4. COSY (blue arrows), HMBC (green arrows) and ROESY (violet arrows) correlations indicating the structures of multiformins E (**10**) – J (15)

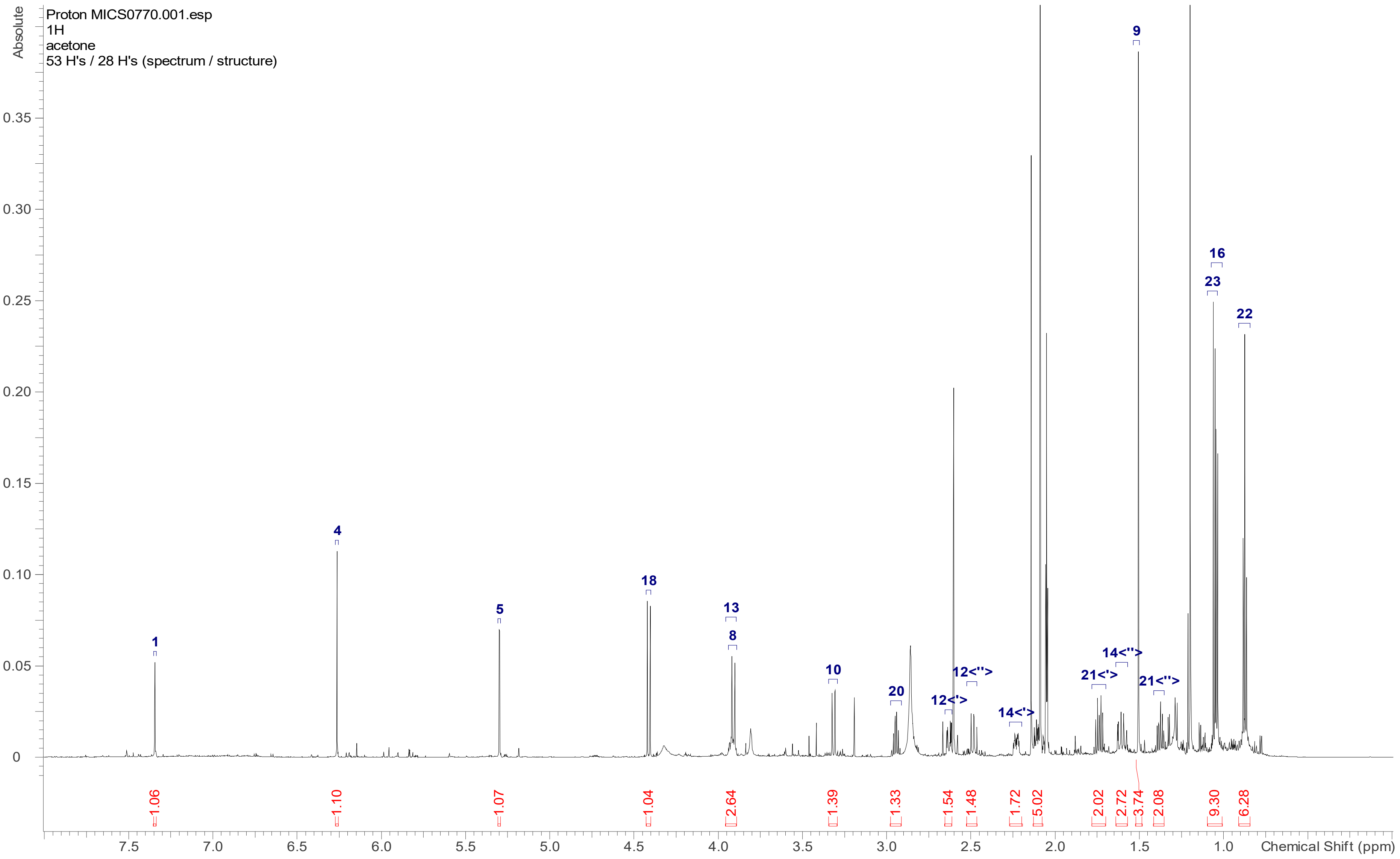

Figure S5. 1H NMR spectrum (700 MHz, acetone-d6) of multiformin E (**10**).

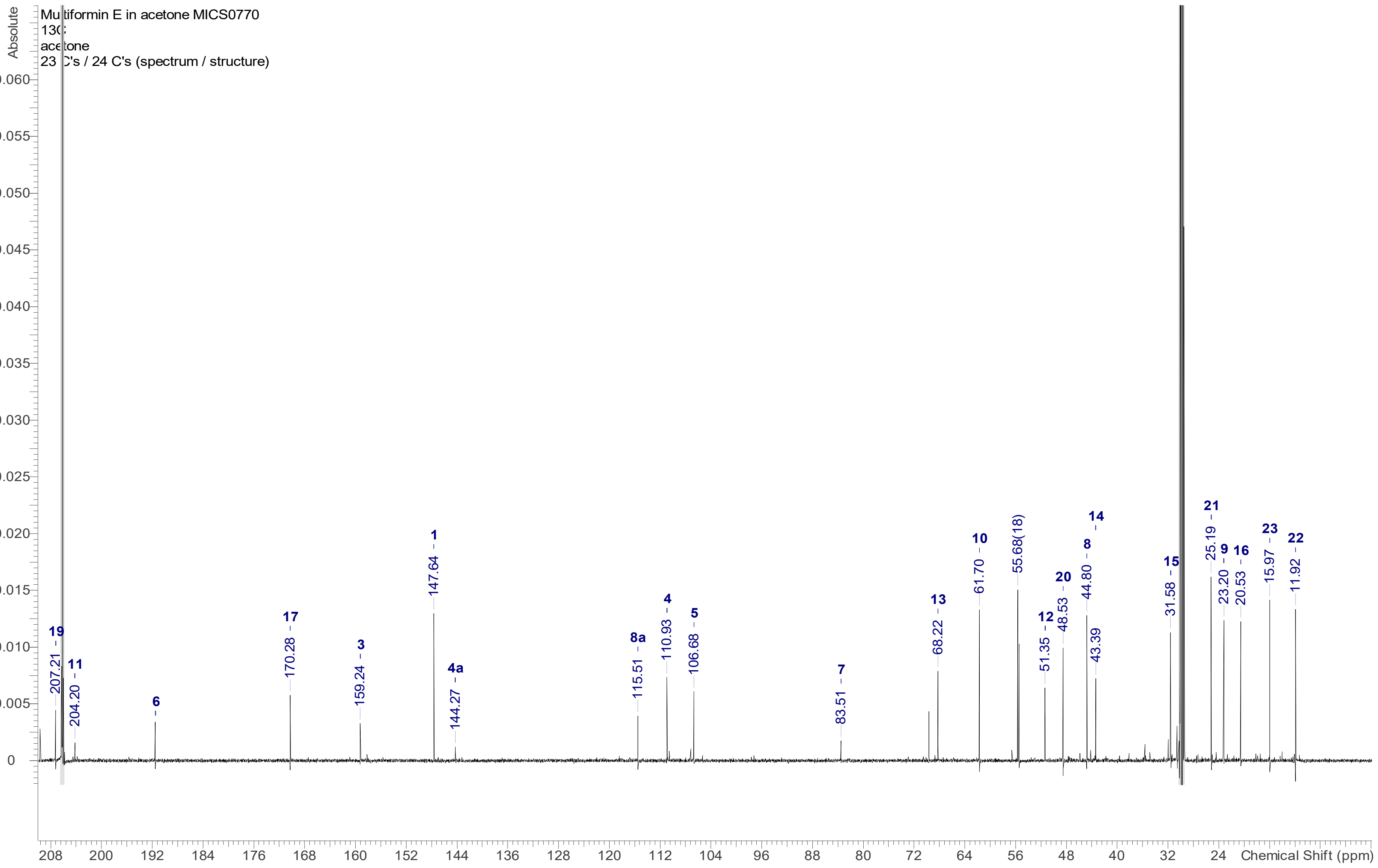

Figure S6. ^{13}C NMR spectrum (175 MHz, acetone-*d*$_6$) of multiformin E (**10**).

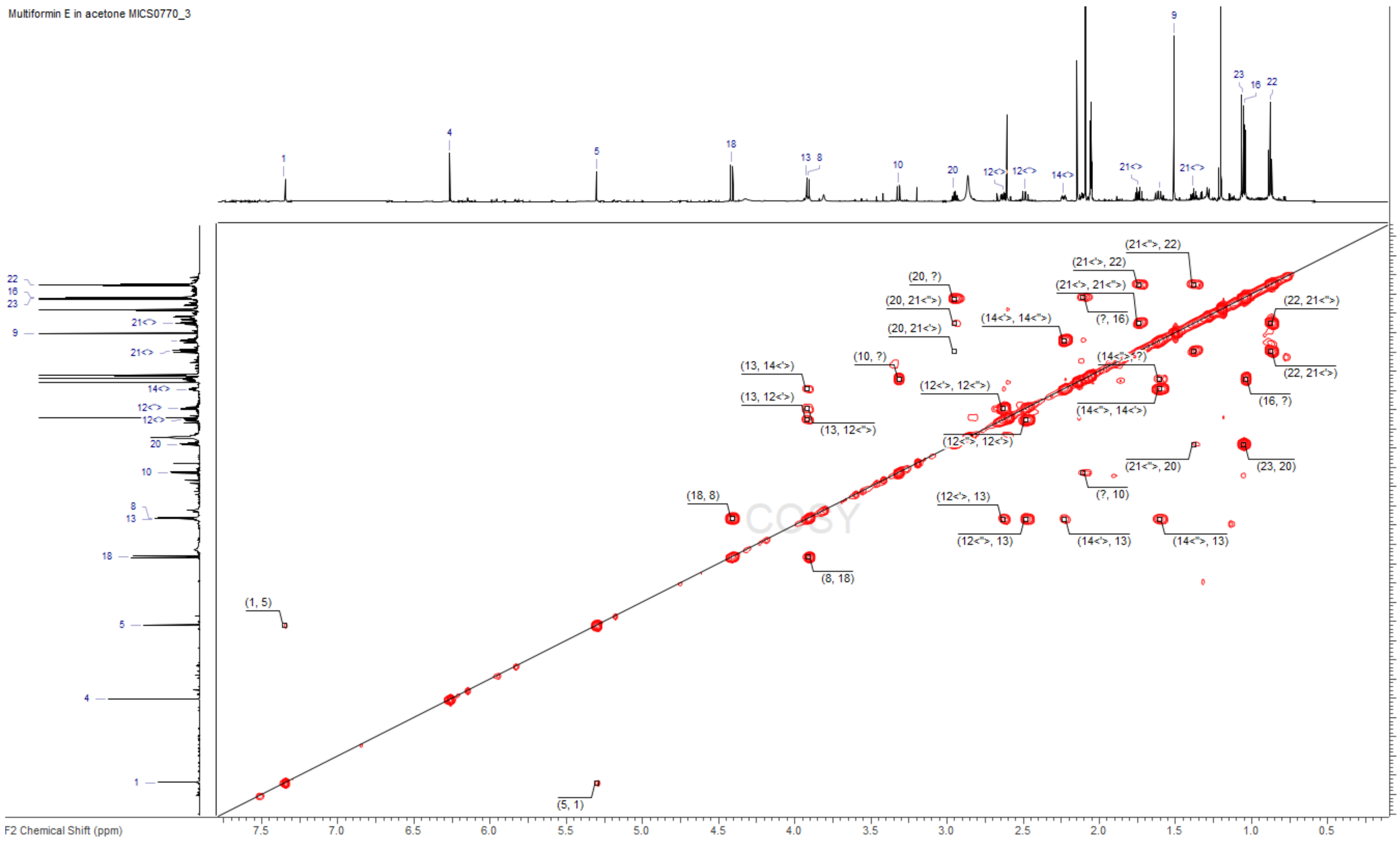

Figure S7. COSY NMR spectrum (700 MHz, acetone-d_6) of multiformin E (**10**).

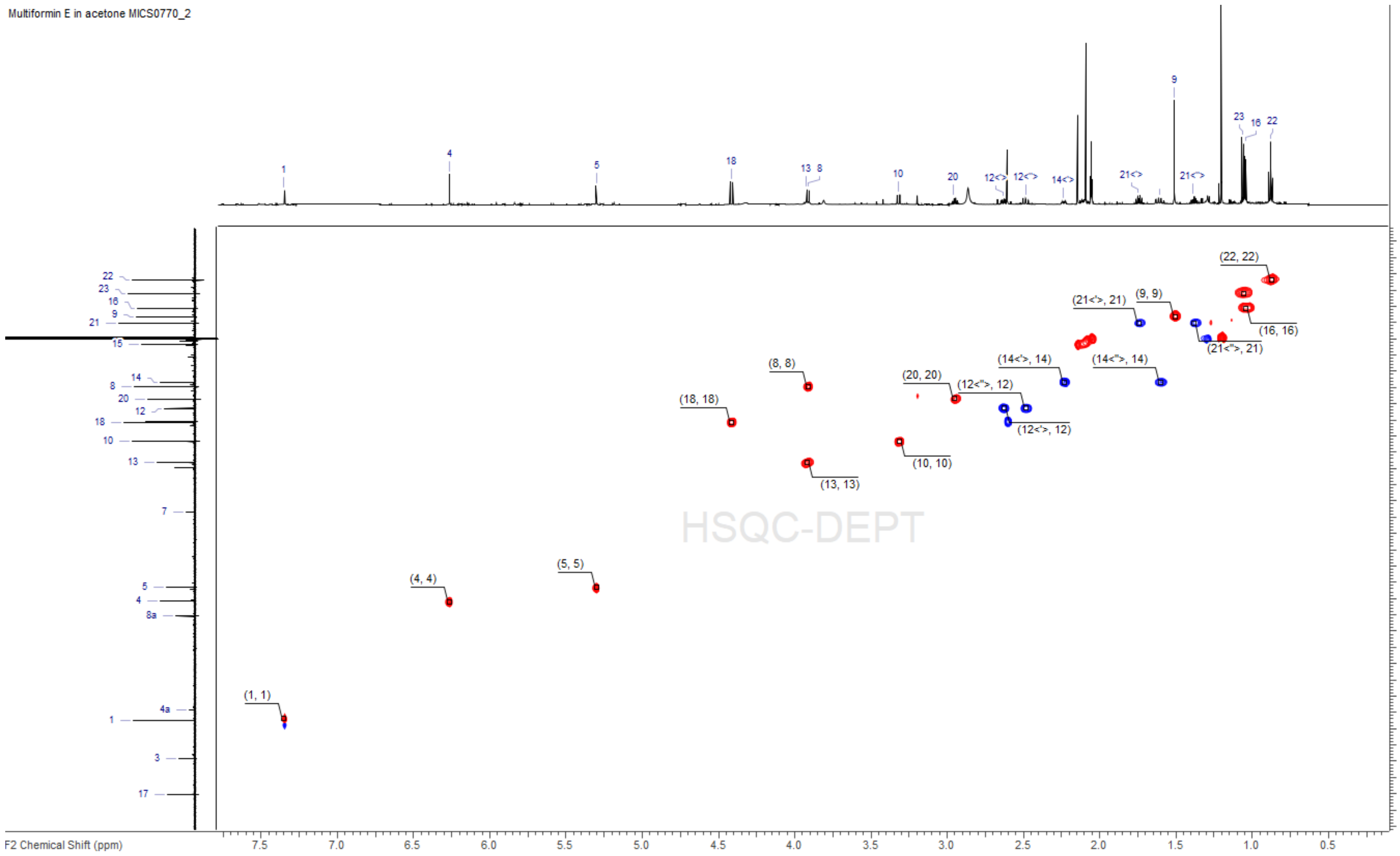

Figure S8. HSQC NMR spectrum (700 MHz, acetone-d_6) of multiformin E (**10**).

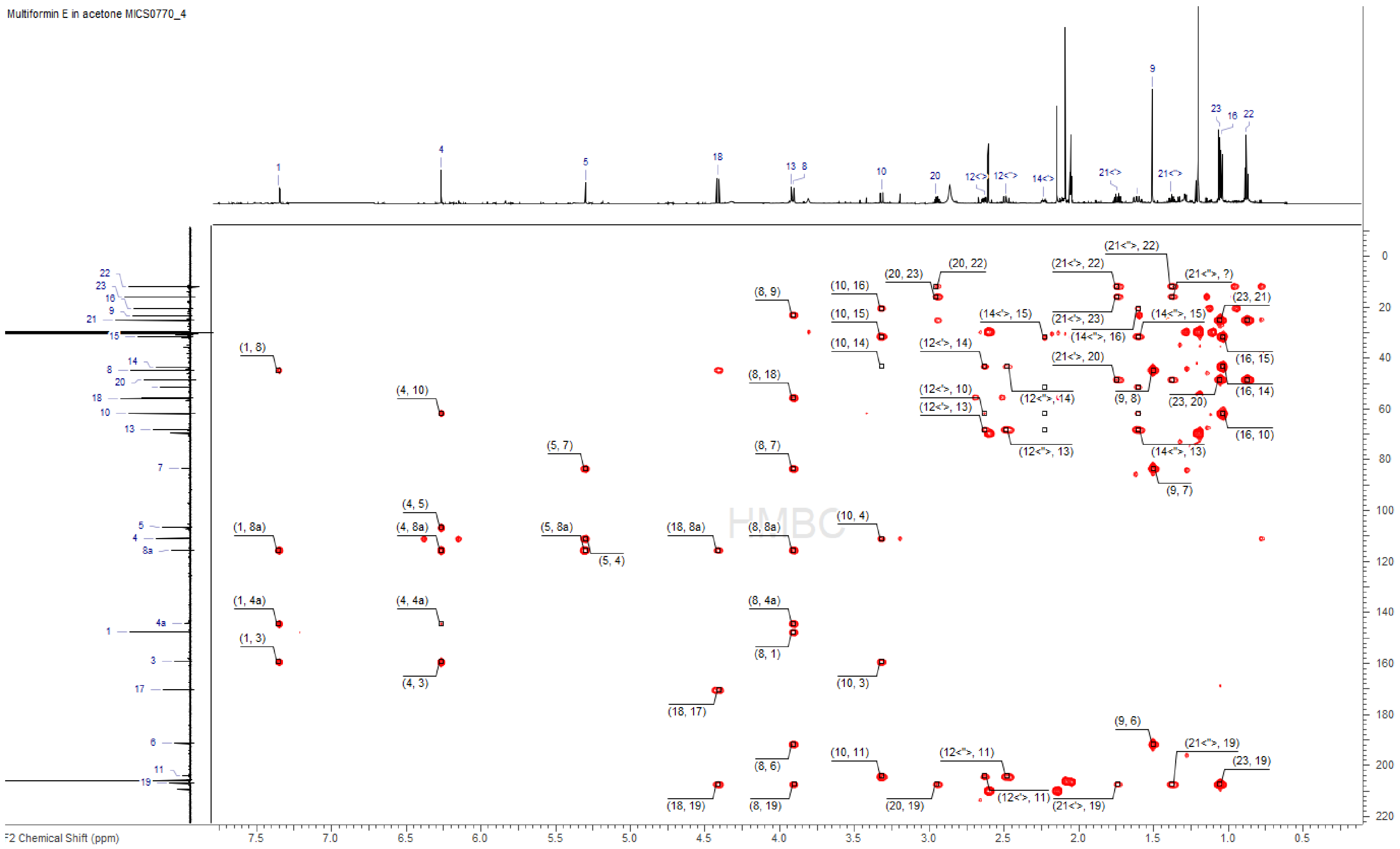

Figure S9. HMBC NMR spectrum (700 MHz, acetone-d_6) of multiformin E (**10**).

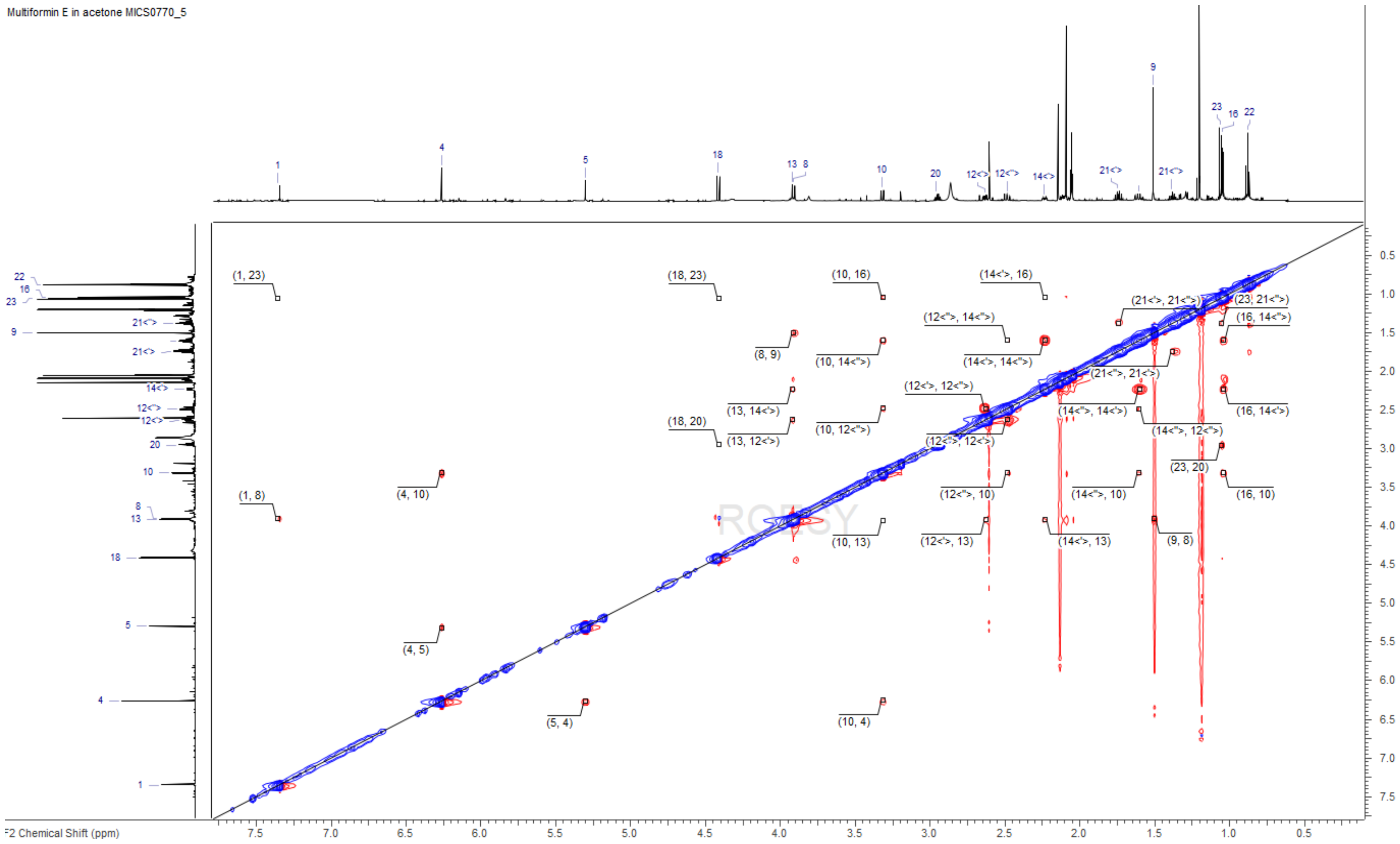

Figure S10. ROESY NMR spectrum (700 MHz, acetone-d_6) of multiformin E (**10**).

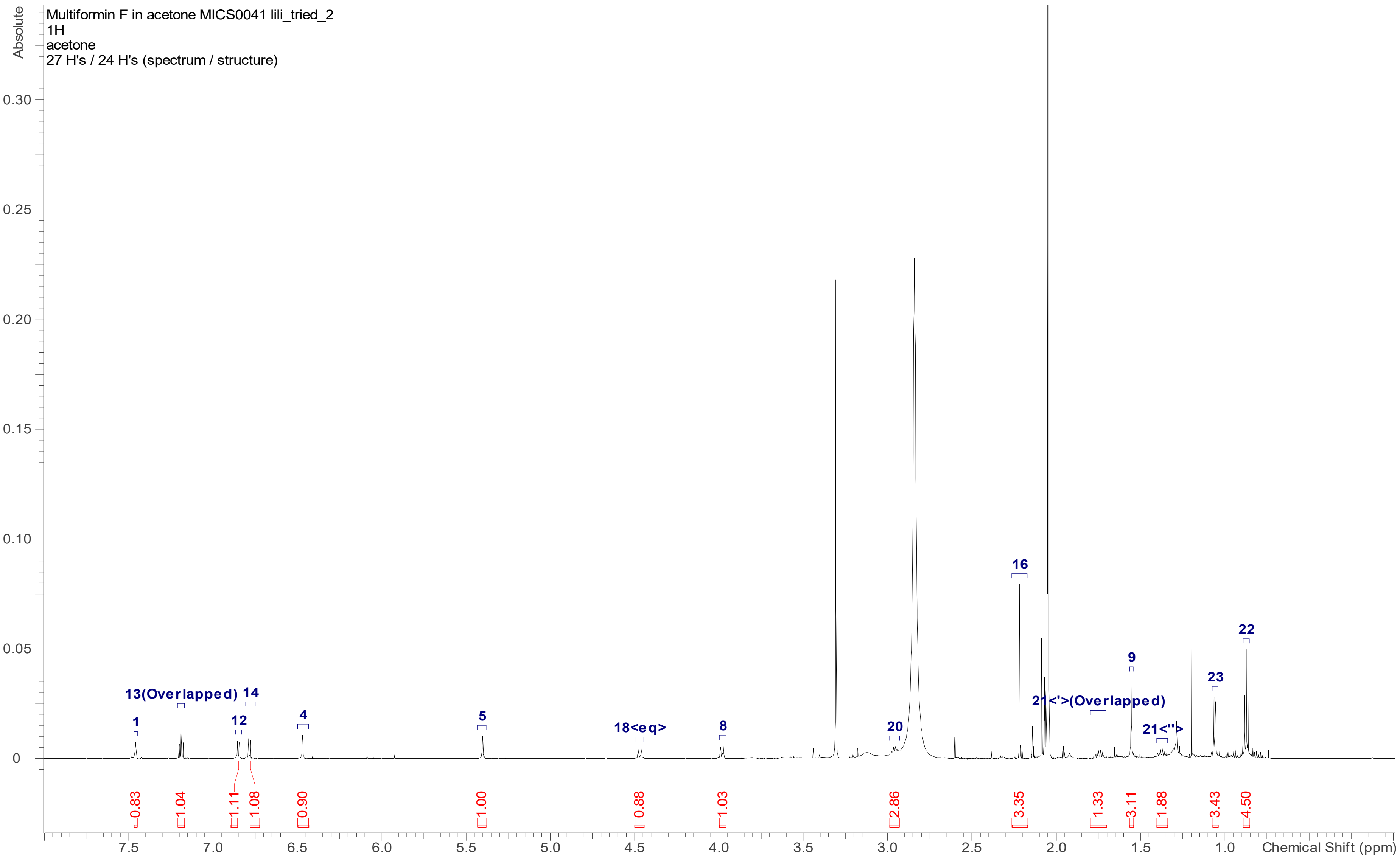

Figure S11. ^{1}H NMR spectrum (700 MHz, acetone-d_6) of multiformin F (**11**).

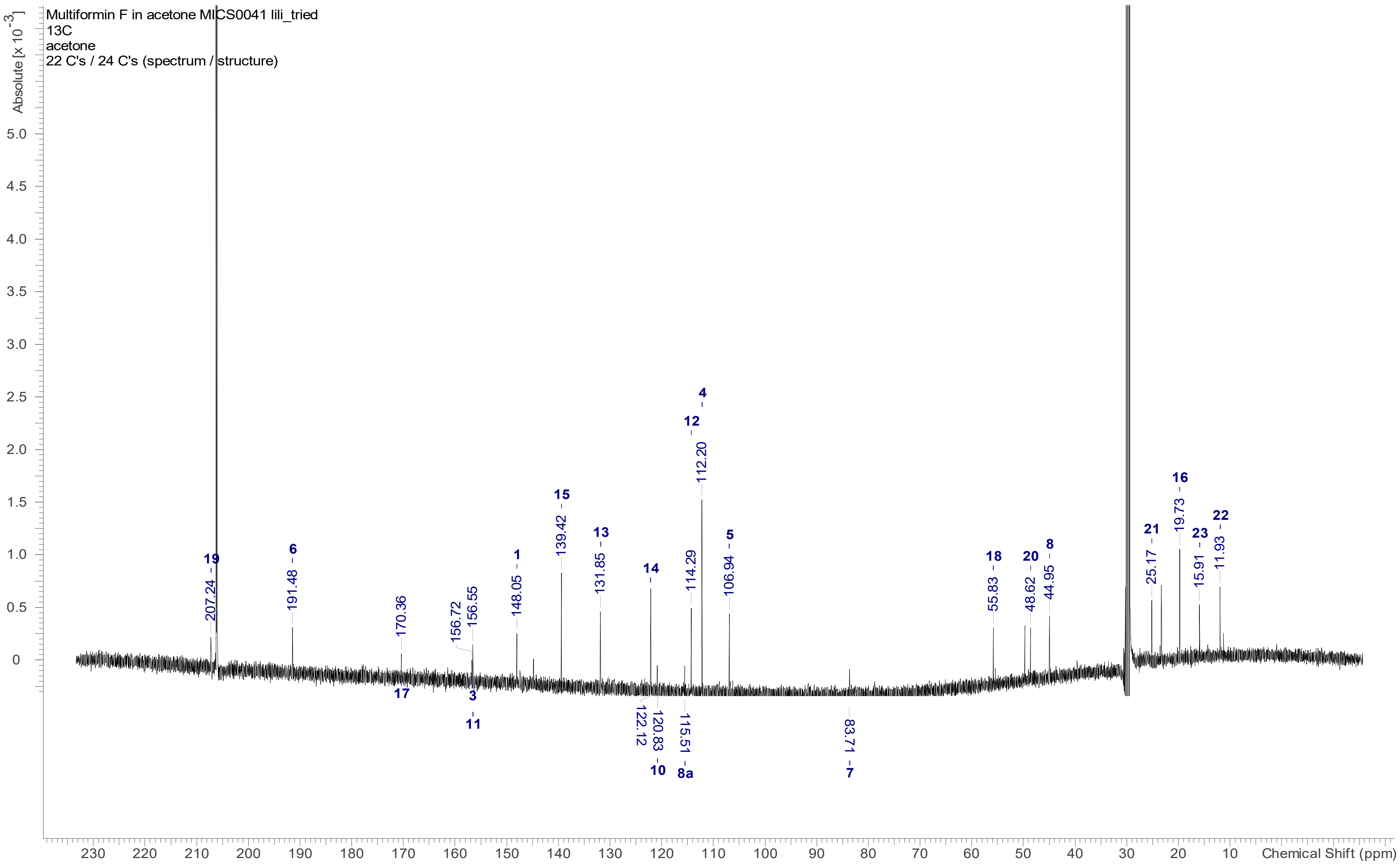

Figure S12. ^{13}C NMR spectrum (175 MHz, acetone-d_6) of multiformin F (**11**).

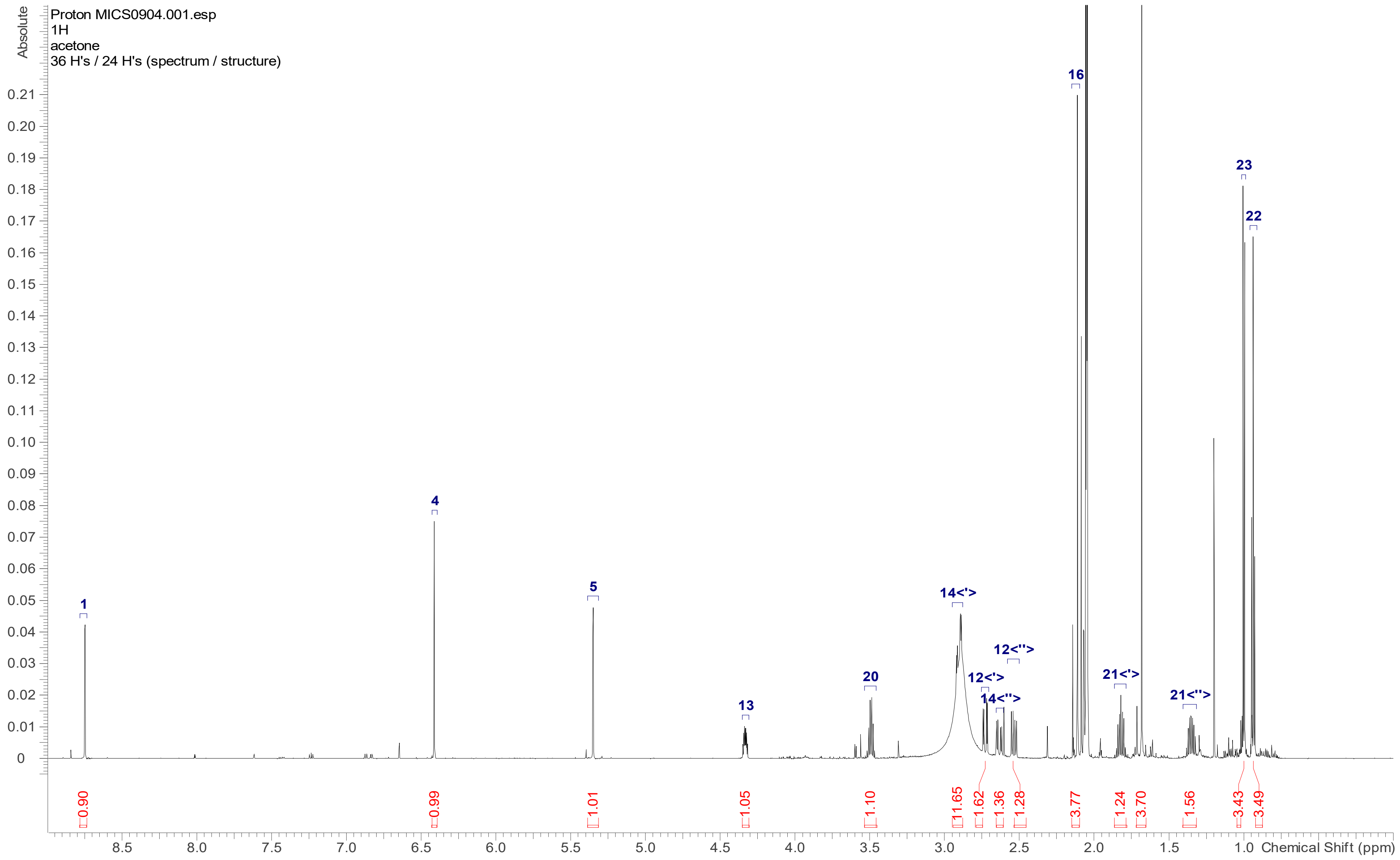

Figure S13. ^{1}H NMR spectrum (700 MHz, acetone-d_6) of multiformin G (**12**).

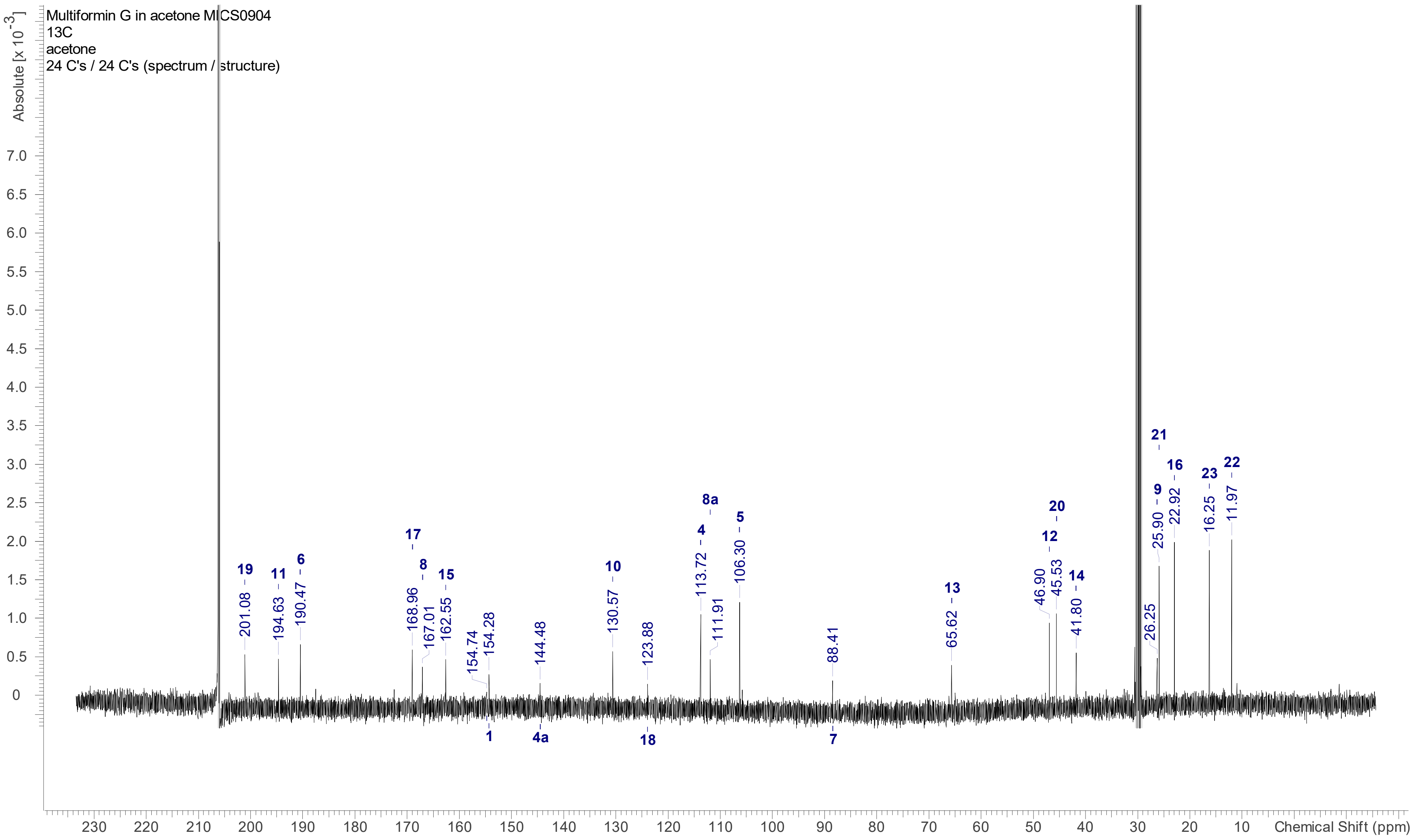

Figure S14. ^{13}C NMR spectrum (175 MHz, acetone-d_6) of multiformin G (**12**).

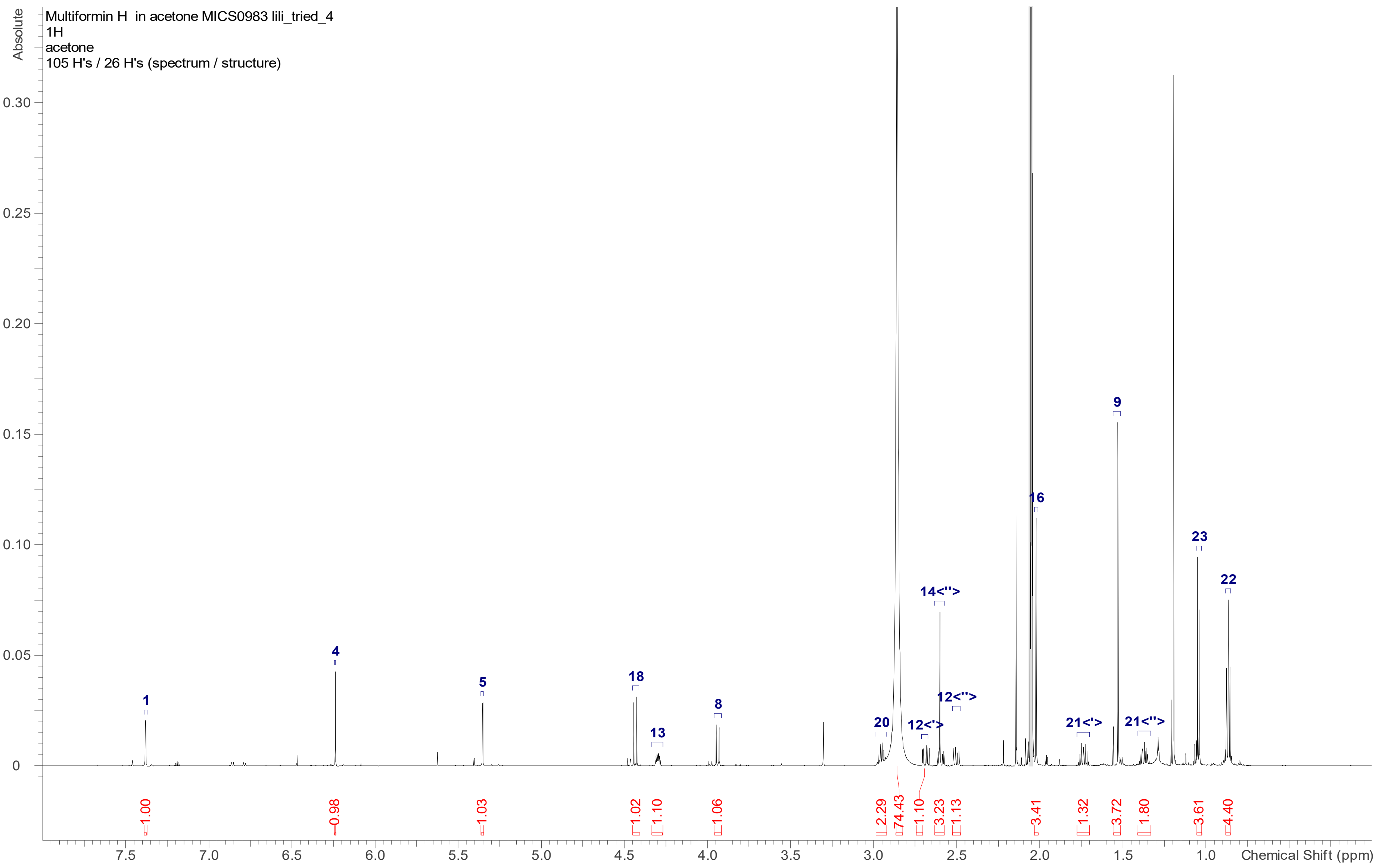

Figure S15. ¹H NMR spectrum (700 MHz, acetone-d_6) of multiformin H (**13**).

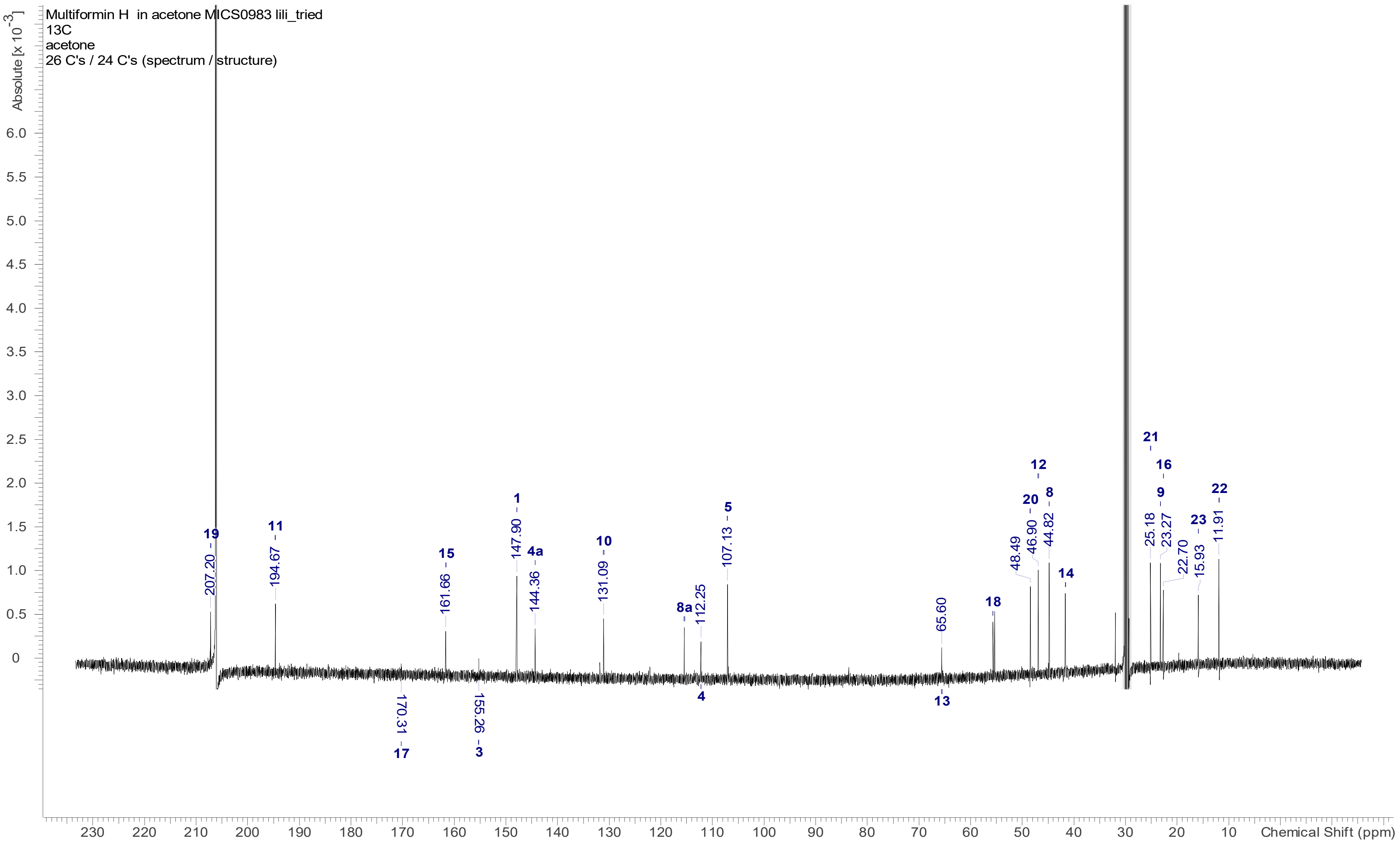

Figure S16. ¹³C NMR spectrum (175 MHz, acetone-d_6) of multiformin H (**13**).

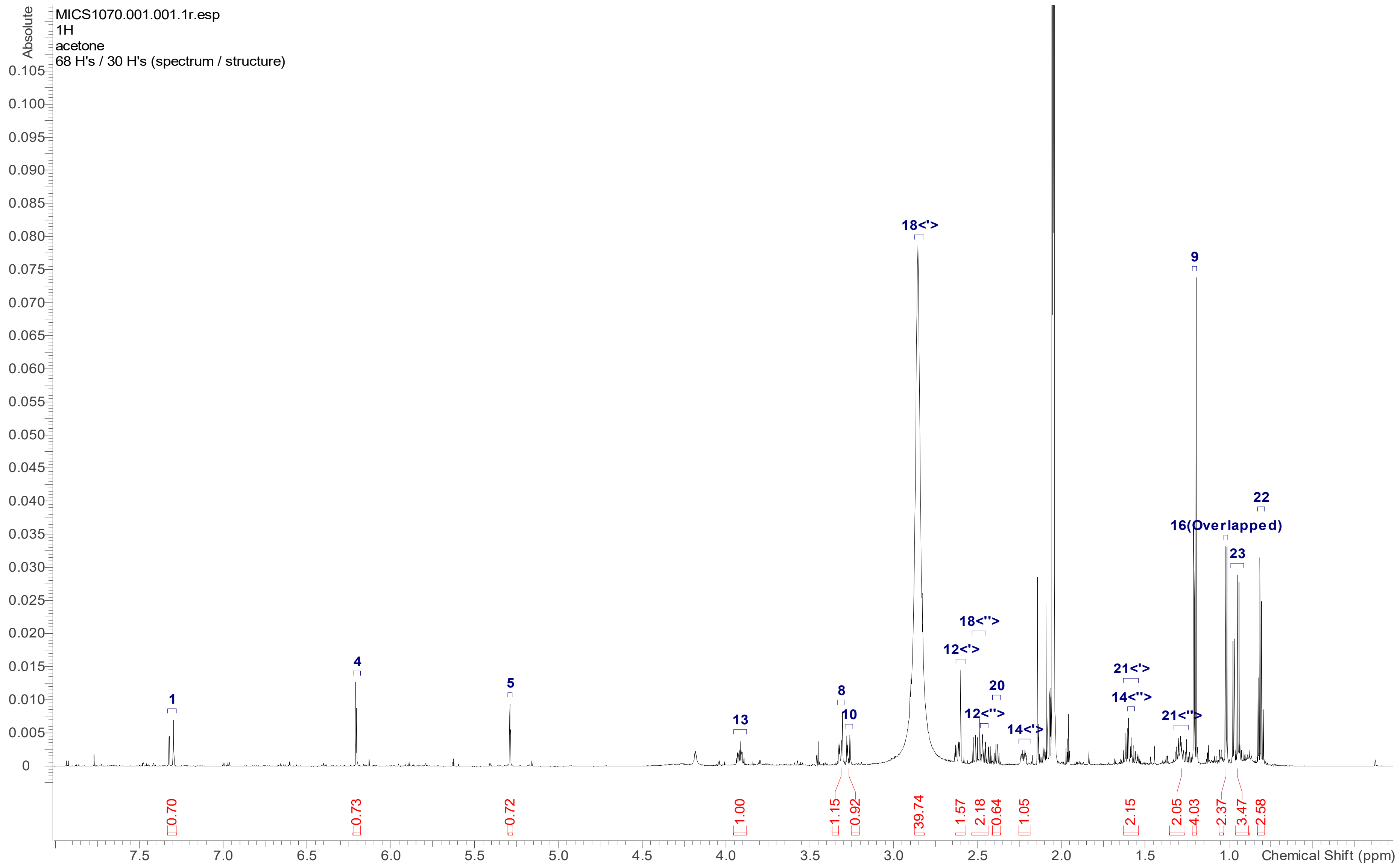

Figure S17. ^{1}H NMR spectrum (700 MHz, acetone-d_6) of multiformin I (**14**).

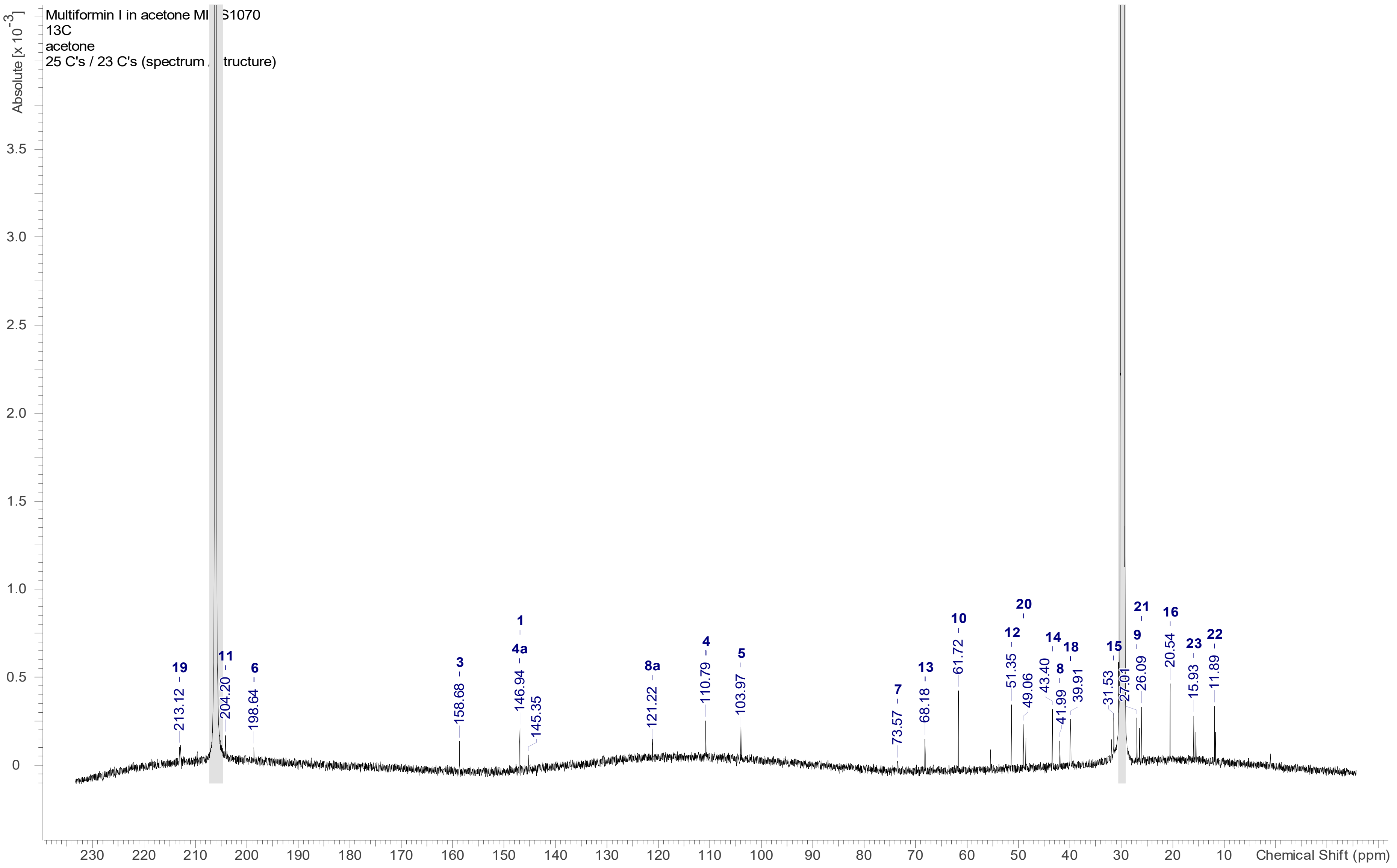

Figure S18. ^{13}C NMR spectrum (175 MHz, acetone-d_6) of multiformin I (**14**).

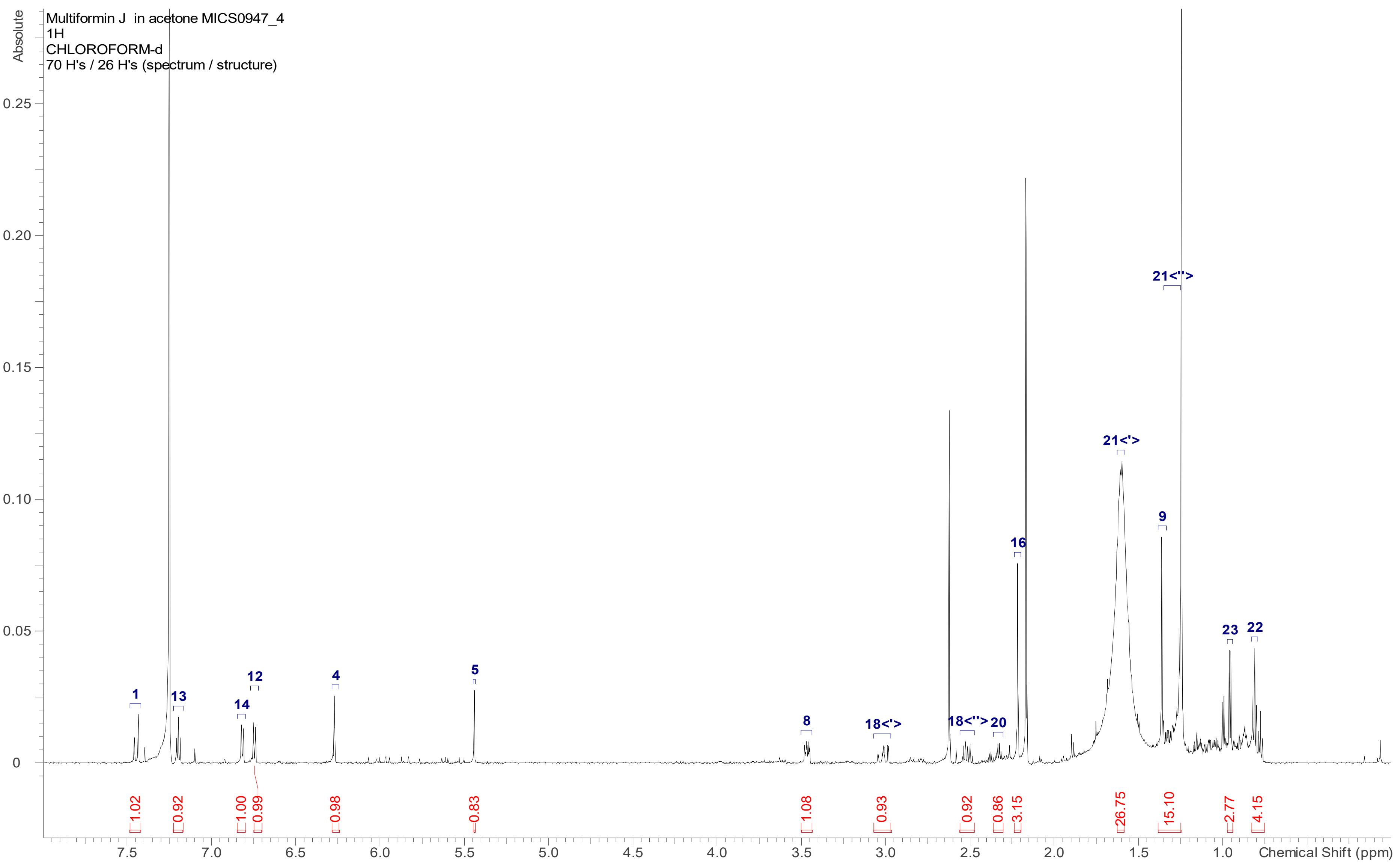

Figure S19. ^{1}H NMR spectrum (700 MHz, $CDCl_3$-*d*) of multiformin J (**15**).

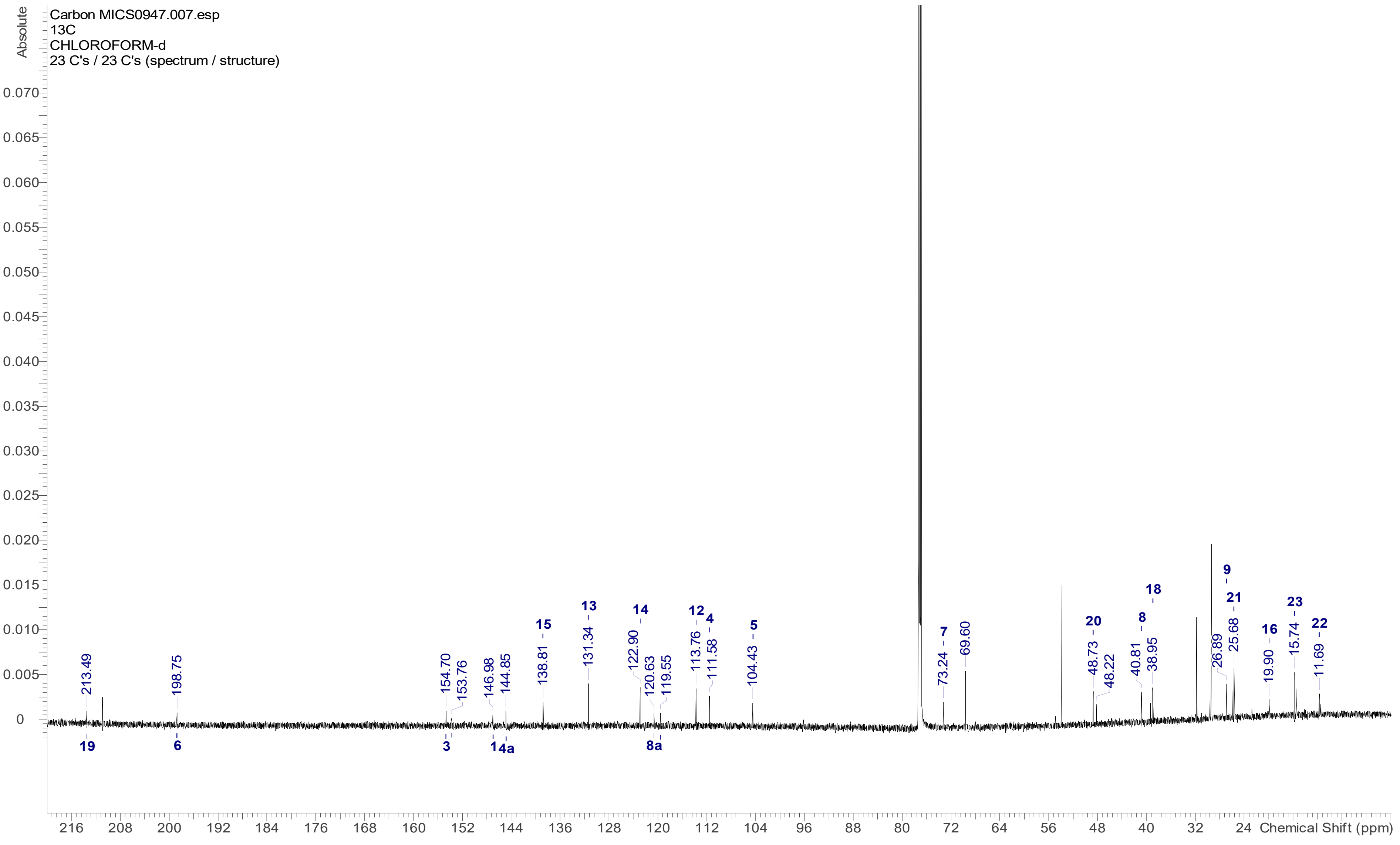

Figure S20. ^{13}C NMR spectrum (175 MHz, $CDCl_3$-*d*) of multiformin J (**15**).

rubiginosin A (1

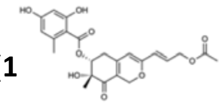

rubiginosin C (20)

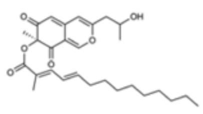

mitorubin (21)

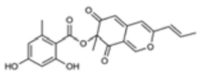

mitorubinol (22)

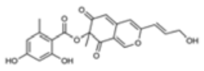

fragirubin F (23)

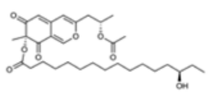

bepridil (24)

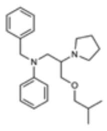

diltiazem (25)

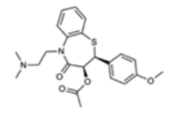

pimozide (26)

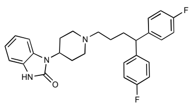

trifluoperazine (27

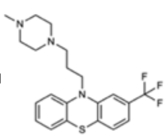

Bay K8644 (28)

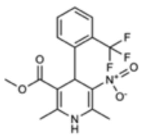

methoxyverapamil (29)

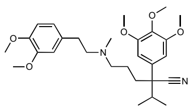

nifedipine (30)

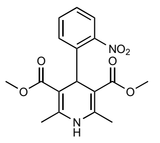

flunarizine (31)

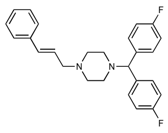

Figure S21. Compounds tested in the microarray-based assay.

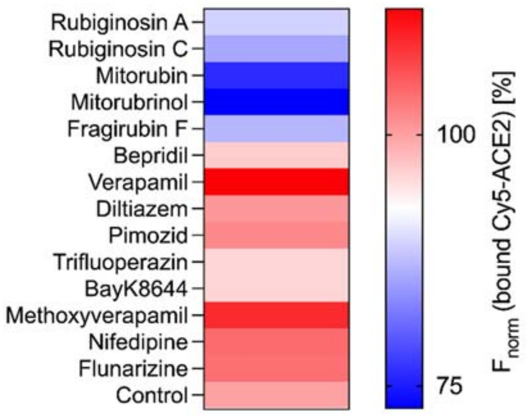

Figure S22. Heat map showing binding inhibition of ACE2 and spike protein by substances **19-31**. The fluorescence intensity is given in percentage compared to Cy5-ACE2 binding in control without substance

Table S1. Cytotoxicity of cohaerins (**1-5**) and multiformins (**6-15**) tested on mouse fibroblast L-929 cancer cells and KB3.1 ACC 158 cells. *no cytotoxic activity, **no cytotoxic activity, only inhibition of proliferation.

No.	Name	Cytotoxicity mouse fibroblast L-929 cancer cells (µg/mL) [30]	Cytotoxicity KB3.1 ACC 158 cells (IC50 µg/mL)
1	Cohaerin C	9.8	20
2	Cohaerin D	1.5	n.d.
3	Cohaerin E	7.0	n.d.
4	Cohaerin F	0.6	17
5	Cohaerin G	>10	2.4
6	Multiformin A	n.d.	3.0
7	Multiformin B	n.d.	17
8	Multiformin C	n.d.	23
9	Multiformin D	n.d.	*
10	Multiformin E	n.d.	30
11	Multiformin F	n.d.	7.4
12	Multiformin G	n.d.	*
13	Multiformin H	n.d.	24**
14	Multiformin I	n.d.	**
15	Multiformin J	n.d.	n.d.

Table S2. All tested substances in the microarray-based screening assay. Inhibition of ACE2 and spike protein binding is given in percentage based on fluorescence signal of control without substance.

No.	Name	Concentration (µM)	Inhibition (%)
1	Cohaerin C	40	24.98
2	Cohaerin D	40	45.05
3	Cohaerin E	40	35.96
4	Cohaerin F	40	55.65
5	Cohaerin G	40	−2.04
6	Multiformin A	40	18.10
7	Multiformin B	40	1.94
8	Multiformin C	40	23.53
9	Multiformin D	40	16.44
10	Multiformin E	40	n.d.
11	Multiformin F	40	5.19
12	Multiformin G	40	13.39
13	Multiformin H	40	−9.45
14	Multiformin I	40	−15.05
15	Multiformin J	40	31.40

16	Mycophenolic acid (MPA)	40	25.31
17	Imatinib	40	−9.45
18	Quinacrine dihydrochloride (QNHC)	40	20.08
19	Rubiginosin A	22	10.88
20	Rubiginosin C	22	14.18
21	Mitorubin	26	23.90
22	Mitorubinol	25	27.18
23	Fragirubin F	18	12.99
24	Bepridil	27	3.40
25	Diltiazem	24	−0.68
26	Pimozid	22	−1.84
27	Trifluoperazin	25	4.06
28	BayK8644	28	4.11
29	Methoxyverapamil	19	−9.32
30	Nifedipine	29	−4.25
31	Flunarizine	25	−3.80
